# AI-Based Prognostic Risk Stratification of Adult-Type Diffuse Glioma Patients from H&E-Stained Slides

**DOI:** 10.3390/cancers18203259

**Published:** 2026-10-09

**Authors:** Shubham Innani, Carla Pitarch-Abaigar, Marwan M. Majeed, Hannah Harmsen, W. Robert Bell, Dimitrios Makris, Spyridon Bakas

**Affiliations:** 1 Division of Computational Pathology, Department of Pathology and Laboratory Medicine, Indiana University School of Medicine, Indianapolis, IN 46202, USA; sinnani@iu.edu (S.I.); cpitarch@iu.edu (C.P.-A.); mmajeed@iuhealth.org (M.M.M.); hharmsen@iuhealth.org (H.H.); rb27@iu.edu (W.R.B.); 2 Indiana University Melvin and Bren Simon Comprehensive Cancer Center, Indianapolis, IN 46202, USA; 3 Department of Computer Science, Kingston University, London KT1 2EE, UK; d.makris@kingston.ac.uk; 4 Department of Neurological Surgery, Indiana University School of Medicine, Indianapolis, IN 46202, USA; 5 Department of Computer Science, Luddy School of Informatics, Computing, and Engineering, Indiana University, Indianapolis, IN 46202, USA; 6 Department of Biostatistics and Health Data Science, Indiana University School of Medicine, Indianapolis, IN 46202, USA; 7 Department of Radiology and Imaging Sciences, Indiana University School of Medicine, Indianapolis, IN 46202, USA

**Keywords:** computational pathology, glioma, survival prediction, foundation models, multiple instance learning, risk stratification, whole-slide imaging

## Abstract

**Background/Objectives:** Adult-type diffuse gliomas (ADGs) are aggressive brain tumors with highly variable clinical outcomes. Prognostic assessment directly from routine hematoxylin and eosin (H&E)-stained whole-slide images (WSIs) could support personalized decision-making. **Methods:** To our knowledge, this is the first systematic benchmarking of pathology foundation models (FMs) and multiple instance learning (MIL) methods for overall survival (OS) risk stratification in ADGs under the 2021 World Health Organization (WHO) classification. A retrospective, multi-institutional cohort of 817 subjects from The Cancer Genome Atlas (TCGA) was analyzed. Following tissue segmentation and patch-level curation, we evaluated 77 pathology FM-MIL combinations (11 FMs × 7 MIL methods), together with seven ResNet-50 baseline configurations (84 encoder-MIL configurations in total), using patient-level 10-fold cross-validation. A perturbation-based evaluation assessed attention mechanism faithfulness. **Results:** UNI2 paired with MambaMIL achieved the highest cross-validated concordance index (C-index = 0.77), whereas its time-dependent AUC was modest (0.60–0.61 at 12–36 months), and several combinations performed comparably. Kaplan–Meier analysis demonstrated significant separation between high- and low-risk groups (log-rank *p* = 9.16 × 10^−17^; hazard ratio [HR] = 2.90, 95% confidence interval [CI]: 2.23–3.76), with a median OS of 16.6 months (95% CI: 15.0–20.4) versus 63.5 months (95% CI: 43.9-NR), respectively. Tertile stratification confirmed a monotonic OS gradient from 94.5 months to 14.7 months (*p* = 3.25 × 10^−17^). Risk stratification within individual WHO 2021 types was not significant. Most domain-specific FMs outperformed the ImageNet-pretrained baseline, and the removal of highly attended patches reduced performance, supporting attention faithfulness. **Conclusions:** The AI-derived risk score remained associated with OS after adjustment for individual clinical and molecular variables, but independent prognostic value was not demonstrated in the fully adjusted model (HR = 1.32, 95% CI: 0.94–1.85, *p* = 0.104), and its discrimination largely reflects differences between WHO 2021 types. This benchmarking framework provides guidance for FM-MIL design in computational pathology applications targeting survival prediction; external validation is required before clinical application.

## Simple Summary

Adult-type diffuse gliomas are brain tumors with widely varying outcomes, and clinicians need better ways to estimate prognosis from routinely collected tissue. This study tested whether artificial intelligence could predict overall survival directly from standard hematoxylin and eosin-stained whole-slide images. We compared 77 pathology foundation model–multiple instance learning combinations, together with seven ImageNet-pretrained ResNet-50 baseline configurations, in 817 patients from The Cancer Genome Atlas. In internal cross-validation, the best-performing model separated patients into groups with markedly different survival. However, much of this separation reflected differences between the major tumor types, and the model did not show independent prognostic value once all established clinical and molecular factors were considered together. We also tested whether the model’s attention scores identified image regions that influenced its predictions and found that removing highly attended regions reduced performance. These results provide practical guidance for designing pathology-based survival models and indicate that external validation is required before artificial intelligence can be considered as a complementary prognostic tool in diffuse glioma.

## 1. Introduction

Adult-type diffuse gliomas (ADGs) are infiltrative brain tumors with highly heterogeneous clinical, histopathologic, and molecular profiles contributing to variable patient outcomes and an overall poor prognosis, particularly in high-grade forms [1]. The 2021 WHO Classification of Central Nervous System (CNS) Tumors refined glioma diagnostics by integrating histologic features with molecular markers, dividing ADGs into three types: (i) oligodendroglioma, isocitrate dehydrogenase (IDH)-mutant, 1p/19q-codeleted (Grades 2–3); (ii) astrocytoma, IDH-mutant (Grades 2–4); and (iii) glioblastoma, IDH-wildtype (Grade 4; GBM).

Following the WHO 2021 reclassification of the Cancer Genome Atlas (TCGA) data, Zakharova et al. [2] quantified inter-subtype survival differences using Cox proportional hazards (CoxPH) models. Specifically, oligodendrogliomas (Grade 2) carry approximately a 2.5-fold lower hazard of death than astrocytomas (Grade 2) (HR ≈ 0.40), while astrocytomas (Grade 4) carry approximately a 3-fold lower hazard than GBMs (Grade 4) (HR ≈ 0.33). Throughout this study, we use overall survival (OS), time from diagnosis to death or last follow-up, as the primary clinical endpoint. Despite these molecular refinements, considerable within-subtype variability in clinical outcomes persists [3], underscoring the need for individualized, quantitative prognostic tools.

Accurate risk stratification can directly influence clinical decision-making, helping to tailor therapy intensity, reduce aggressive treatment for lower-risk patients, and identify those likely to benefit from intensive or novel interventions [4]. While expert neuropathologic assessment remains the frontline source of prognostic information, traditional microscopic evaluation is inherently limited by observer variability. Artificial intelligence (AI) is increasingly applied across neurosurgery and neuro-oncology, supporting diagnosis, prognostication, clinical decision-making, and perioperative care [5]. Advances in digital pathology and computational imaging have shown promise for data-driven prognostic stratification in both radiology [6] and pathology [7,8,9,10,11] by capturing extensive visual and sub-visual cues. AI-based approaches [12,13] can extract histomorphological features [14] associated with patient prognosis, augmenting traditional pathology with data-driven insights.

Building on progress in computational pathology for WSI analysis, weakly supervised learning bridges the limitations of supervised and unsupervised paradigms by leveraging patient-level survival outcomes rather than detailed region-level labels [15]. In this framework, gigapixel WSIs are divided into patches, features are extracted per patch, and patch-level information is aggregated into slide-level representations correlated with clinical outcomes. This strategy has enabled accurate classification and prognostic modeling across diverse applications [12,16,17].

Recent advances in pathology foundation models (FMs), pretrained on large-scale WSI datasets, have substantially improved representation learning in histopathology [18,19,20]. These models generate high-dimensional embeddings that can be aggregated by MIL methods for slide-level prediction [21,22]. Such approaches have been benchmarked successfully in classification tasks [23,24,25], yet systematic benchmarking of FM-MIL combinations specifically for survival prediction in ADGs remains limited. As the landscape of FMs and MIL methods continues to evolve, there is a pressing need for comprehensive benchmarking to evaluate their relative strengths and limitations for prognostic applications.

Previous studies of survival prediction from glioma histology have established feasibility but leave several questions open. Mobadersany et al. [26] predicted outcome from pathologist-selected regions of H&E images, alone and combined with genomic markers, and Steyaert et al. [27] combined WSIs with gene expression data in adult and pediatric brain tumors; both studies preceded the WHO 2021 classification. Chen et al. [28] integrated histology and genomics across 14 cancer types, including TCGA gliomas, using ImageNet-pretrained features and a single attention-based MIL design, and Chunduru et al. [4] applied neural network-based Cox regression to glioma WSIs. Consequently, it remains unclear (i) whether pathology-specific FMs improve survival prediction over ImageNet-pretrained encoders in ADG defined by the WHO 2021 criteria, (ii) how much performance depends on the encoder versus the MIL aggregator, (iii) whether attention scores reflect the regions that drive predictions, and (iv) to what extent a morphology-derived risk score captures prognostic information beyond the WHO 2021 types themselves. A controlled comparison of 11 FMs and seven MIL methods on identical patient-level folds addresses the first two questions directly and provides a common reference for the other two. We therefore regard this work primarily as a methodological benchmark whose clinical implications require confirmation in independent cohorts.

The objectives of this study are: (i) to develop and benchmark a weakly supervised deep learning framework for OS risk stratification in ADG from H&E-stained WSIs under the WHO 2021 criteria; (ii) to provide, to our knowledge, the first comprehensive evaluation of pathology FM-MIL combinations (77 combinations: 11 FMs × 7 MIL methods) with a ResNet-50 baseline evaluated using the same seven MIL methods for survival prediction in ADG; (iii) to introduce a perturbation-based evaluation of attention mechanism faithfulness; and (iv) to contextualize AI-derived risk scores within a CoxPH framework against key WHO 2021 molecular and clinical variables.

Regarding intended use, the principal question addressed here is whether a morphology-derived risk score obtained from routine H&E slides can stratify OS across the full spectrum of ADG, as encountered at histopathologic assessment and before molecular results become available. A secondary, exploratory question is whether the same score adds prognostic information among patients who already share a WHO 2021 diagnosis. Because oligodendroglioma, IDH-mutant astrocytoma, and IDH-wildtype glioblastoma have markedly different survival distributions, a model that recognizes their morphology can achieve a relatively high C-index without resolving clinically relevant prognostic heterogeneity within each type. We therefore report within-type and multivariable analyses alongside the pooled results and interpret the pooled discrimination accordingly.

## 2. Materials and Methods

### 2.1. Data

We identified a retrospective, multi-institutional cohort of 1628 diagnostic formalin-fixed paraffin-embedded (FFPE) H&E-stained WSIs (817 subjects) with OS data from the TCGA-LGG and TCGA-GBM collections. Since these collections comprise subjects diagnosed under the 2000 or 2007 WHO classification, board-certified neuropathologists reviewed available clinical, histologic, and molecular data and reclassified cases according to the WHO 2021 schema as annotated by Zakharova et al. [2]. Characteristics considered included IDH mutation status, 1p/19q codeletion, ATRX, TERT promoter mutations, EGFR amplification, CDKN2A/B homozygous deletion, chromosome 7 gain/10 loss, and H3 K27 and G34 mutations. Subjects lacking sufficient molecular data, or not mappable to one of the three ADG categories (e.g., H3-mutant diffuse gliomas, anaplastic astrocytoma with piloid features, or PXA variants with BRAF mutation and CDKN2A/B loss), could not be confidently assigned a WHO 2021 diagnosis and were excluded from all analyses that required a WHO 2021 label. Because the training target was OS, which does not depend on the diagnostic label, all 817 subjects (1628 WSIs) were used for model development and for the pooled cross-validated performance estimates. The 656 subjects (1322 WSIs) who were confidently reclassified under WHO 2021 criteria constitute the clinical cohort used for all subgroup, CoxPH, and multivariable analyses; the remaining 161 subjects (306 WSIs) had OS data but no confident WHO 2021 diagnosis. The cohort selection is summarized in Supplementary Figure S2. The 656 subjects were assigned to one of three ADG categories: (i) oligodendroglioma, IDH-mutant, 1p/19q-codeleted (Grades 2–3; N = 141); (ii) astrocytoma, IDH-mutant (Grades 2–4; N = 226); (iii) glioblastoma, IDH-wildtype (Grade 4; N = 289).

Multiple WSIs were retained per patient where available, because including multiple tissue blocks captures intra-tumoral morphologic heterogeneity that a single slide may not represent. Performance was estimated with patient-level 10-fold cross-validation. Patients were randomly assigned to folds, without stratification by event status, histologic type, grade, submitting institution, or follow-up duration, and all WSIs from the same patient were assigned to the same fold, preventing patient-level data leakage. The identical fold assignment was used for all FM-MIL combinations and for the ResNet-50 baseline, so that all combinations were evaluated on the same patients. In each iteration, nine folds were used for training, and the remaining fold was held out for evaluation. No separate independent test set was available; all performance estimates reported herein are therefore internal cross-validation estimates obtained on held-out folds and should not be interpreted as independent (external) test-set performance. Each WSI was scanned at either 40× (0.2456–0.2533 microns per pixel) or 20× (0.4993–0.504 microns per pixel) maximum magnification and standardized to a 20× reference level by downsampling.

### 2.2. Weakly Supervised Framework

**Figure 1:**
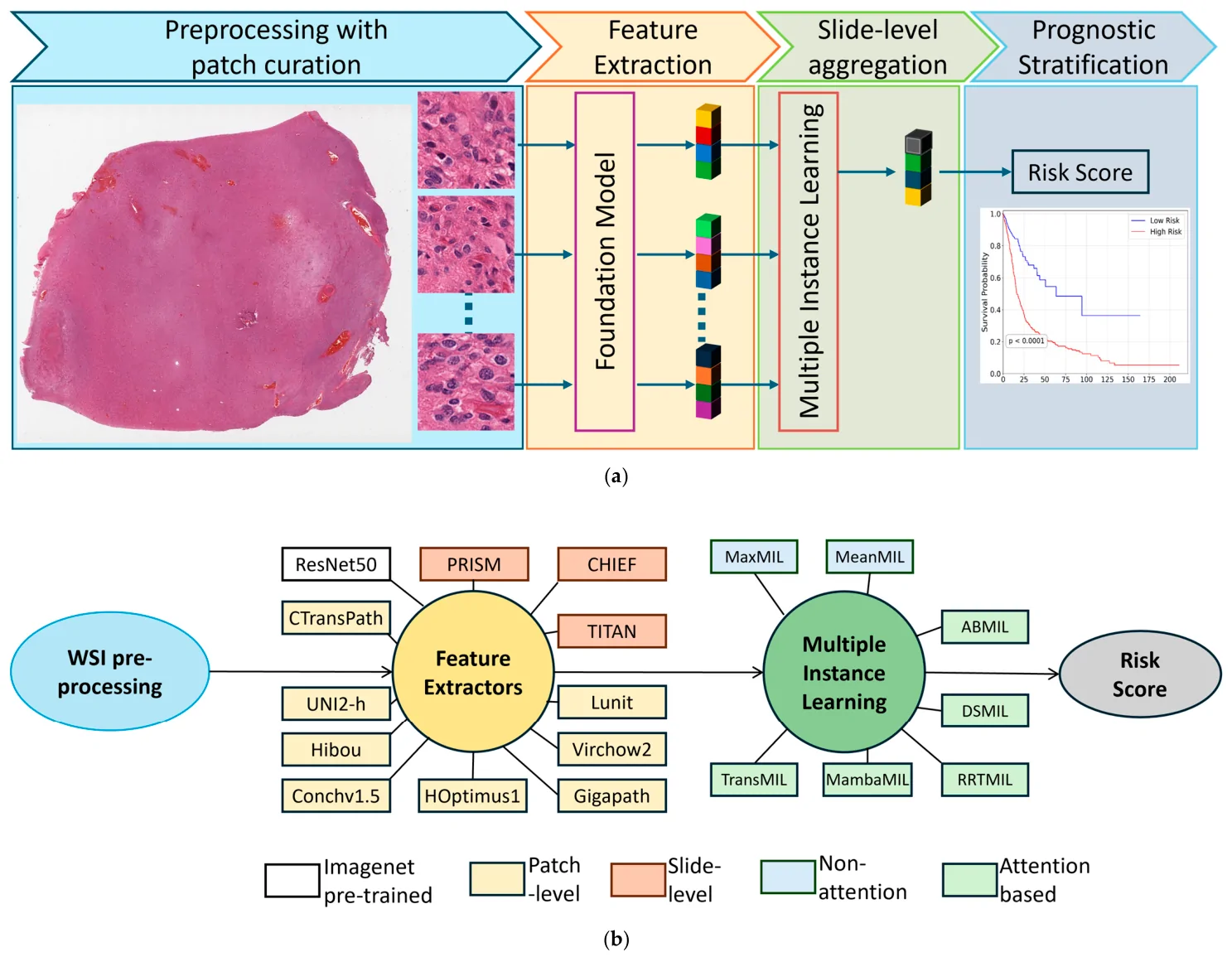

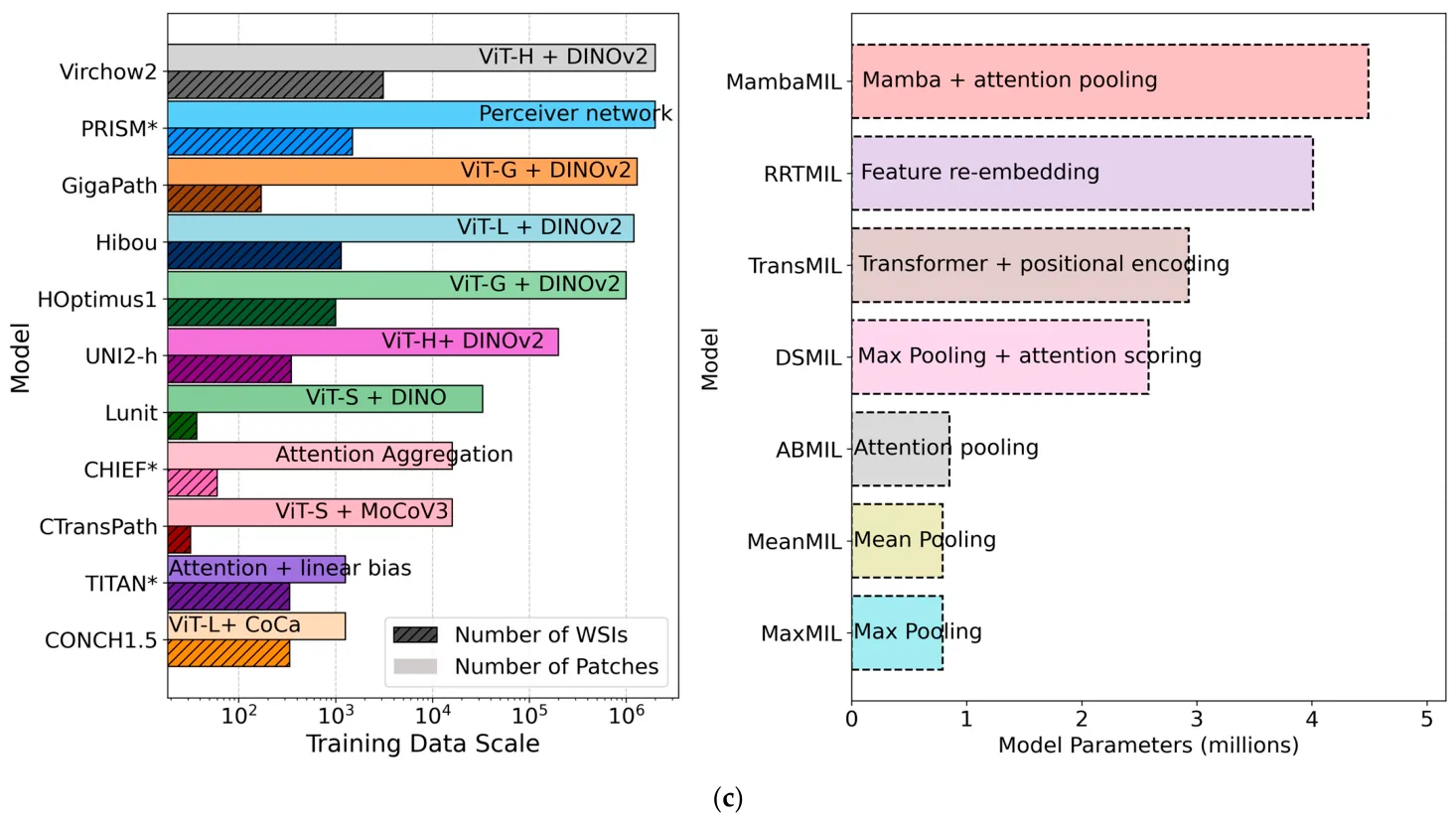
Schematic representation of the proposed methodological workflow. (**a**) Workflow of the weakly supervised framework for patient risk stratification; (**b**) overview of benchmarked FMs and MIL methods; (**c**) comparison of FMs by dataset scale, backbone architecture, and self-supervised learning method (**left**), and comparison of seven MIL methods by aggregation strategy and parameter count (**right**). Asterisks (*) denote slide-level foundation models.

Figure 1a summarizes our weakly supervised pipeline. Tissue segmentation and tiling were performed with the CLAM pipeline [29]. Briefly, a low-resolution thumbnail of each WSI was converted to the hue-saturation-value color space, and the saturation channel was median-filtered (kernel size 7), binarized (threshold 8), and processed with morphological closing (kernel size 4). Tissue contours and holes were filtered by area using the CLAM default thresholds (a_t_ = 100 and a_h_ = 16, in multiples of a 512 × 512-pixel reference patch; at most 8 holes per contour). Each WSI was then tiled into non-overlapping 256 × 256-pixel patches at 20× magnification (approximately 0.5 microns per pixel, corresponding to a field of view of approximately 128 × 128 microns); for slides scanned at 40×, the corresponding 512 × 512-pixel regions at native resolution were downsampled to 256 × 256 pixels. A patch was retained if at least one of four points located around its center fell within a tissue contour (CLAM four-point criterion). Retained patches were further subjected to automated curation to remove background, out-of-focus tiles, pen marks, and other non-tissue content, following our previously described procedures [11,14]. All retained patches were used without subsampling. Patches were not restricted to tumor regions because no region-level annotations were available and TCGA diagnostic slides are predominantly tumor-bearing. The median number of retained patches per WSI was 10,354.

#### 2.2.1. Feature Extraction

Curated patches were encoded using pathology-specific FMs as frozen feature extractors (no fine-tuning). We evaluated an ImageNet-pretrained ResNet-50 as a general-purpose baseline against 8 patch-level and 3 slide-level domain-specific FMs (Table 1). The patch-level FMs comprised:

(i) CTransPath [30] (~28 M parameters): Hybrid Swin Transformer and CNN architecture pretrained with MoCo-v3 [31] on ~15.6 M patches from 32,000 WSIs. Generates 768-dimensional embeddings.
(ii) Lunit [32] (~22 M parameters): ViT-Small pretrained with DINO [33] on 33 M patches from 37,000 WSIs. Produces 384-dimensional embeddings.
(iii) UNI2 [18] (~681 M parameters): ViT-Huge [34] pretrained with DINOv2 [35] on 200 M patches from 350,000 WSIs. Produces 1536-dimensional embeddings.
(iv) CONCHv1.5 [36] (~307 M parameters): Vision-language ViT-Large trained with iBOT and CoCa [37] on 1.17 M image-caption pairs. Outputs 768-dimensional embeddings.
(v) GigaPath [19] (~1.1 B parameters): ViT-giant [38] pretrained with DINOv2 on 1.3 B patches from 171,000 WSIs. Produces 1536-dimensional embeddings.
(vi) Hibou [39] (~304 M parameters): ViT-Large pretrained with DINOv2 on 1.2 B patches from 1.1 M WSIs. Outputs 1024-dimensional embeddings.
(vii) H-Optimus-1 [40] (~1.1 B parameters): ViT-Giant [38] pretrained with DINOv2 on >500,000 WSIs from public and proprietary datasets. Produces 1536-dimensional embeddings.
(viii) Virchow2 [20] (~632 M parameters): ViT-Huge pretrained with DINOv2 on 2 B patches from 3.1 M WSIs. Produces 2560-dimensional embeddings and is the largest model by pretraining data volume.

The slide-level FMs comprise:

(ix) PRISM [41]: Multimodal generative FM using a perceiver-based encoder [42], generating 1280-dimensional slide-level embeddings.
(x) CHIEF [43]: Slide-level FM built on CTransPath patch embeddings with an attention mechanism [44]; 768-dimensional.
(xi) TITAN [45]: Uses CONCHv1.5 features with attention-with-linear-biases (ALiBi) [46]; 768-dimensional.

**Table 1:** Overview of foundation models used for feature extraction. Slide-level FMs rely on tile-level encoders internally; tile and slide counts are not applicable (NA).

Model	Param (M)	Algorithm	Tiles (M)	Slides (K)	Embed. Size
Patch-level foundation models					
CTransPath [30]	28	Swin Transformer + MoCov3 [31]	16	32	768
Lunit ViT [32]	22	ViT-S + DINO [33]	33	37	384
UNI2 [18]	681	ViT-H + DINOv2 [35]	200	350	1536
CONCHv1.5 [36]	307	ViT-L + CoCa [37]	16	21	768
GigaPath [19]	1135	ViT-G + DINOv2 [35]	1300	171	1536
Hibou [39]	304	ViT-L + DINOv2 [35]	1200	1139	1024
H-Optimus-1 [40]	1135	ViT-G + DINOv2 [35]	NA	500	1536
Virchow2 [20]	632	ViT-H + DINOv2 [35]	2000	3100	2560
Slide-level foundation models					
PRISM [41]	99	Perceiver-based encoder [42]	-	-	1280
CHIEF [43]	<1	Attention encoder [44]	-	-	768
TITAN [45]	48.5	Attention-with-linear-biases [46]	-	-	768

#### 2.2.2. Multiple Instance Learning

We used MIL to aggregate FM patch embeddings into patient-level representations. In MIL, each patient is treated as a “bag” of patch embeddings associated with a patient-level survival outcome, and no patch-level labels are used at any point [47]. For patients with more than one diagnostic WSI, the patch embeddings from all of their WSIs were concatenated into a single bag before aggregation. Each patient therefore contributed exactly one risk score, and slide-to-patient aggregation was performed at the embedding level rather than by combining slide-level predictions. For training, we used the discrete-time negative log-likelihood (NLL) survival loss [48], which accommodates right-censored survival data. Survival times were discretized into k = 4 intervals defined by the quartiles of the survival times of uncensored patients, and each patient was assigned the interval containing their event or censoring time. For each patient, the model outputs interval-specific hazards h_j_ = σ(z_j_), j = 1, …, k, from which the survival function is S_j_ = ∏_l≤j_ (1 − h_l_), with S_0_ = 1. For a patient in interval y with censoring indicator c (c = 1 if censored), the loss is L = −(1 − c)[log S_y−1_ + log h_y_] − c log S_y_. Censored patients therefore contribute only through their predicted survival up to the censoring interval; the formulation does not require the construction of risk sets or a specific handling of tied event times, and the additional weight on uncensored patients was set to zero. The continuous patient-level risk score was defined as R = −Σ_j_ S_j_, so that higher values indicate worse predicted prognosis. The binary, tertile, and quartile risk groups (Section 2.4) were used exclusively for evaluation (Kaplan–Meier curves and HR reporting) and not during model training or optimization.

Seven MIL methods were benchmarked (Figure 1c):

(i) MeanMIL [49] (~0.79 M parameters): Aggregates patch embeddings by arithmetic mean.
(ii) MaxMIL [49] (~0.79 M parameters): Selects the highest-risk patch as the slide-level score.
(iii) ABMIL [50] (~0.85 M parameters): Learnable attention mechanism; weighted sum of patch embeddings through MLP.
(iv) DSMIL [51] (~2.58 M parameters): Dual-stream max-pooling with attention; fuses patch-level and slide-level classifiers.
(v) TransMIL [52] (~2.93 M parameters): Transformer self-attention with Nyström approximation for spatial context modeling.
(vi) RRTMIL [22] (~4.01 M parameters): Region-wise multi-head self-attention with position encoding; aggregated via ABMIL.
(vii) MambaMIL [21] (~4.49 M parameters): SR-Mamba [53] linear-time sequence model for long-range patch dependencies.

All MIL aggregation layers were trained from scratch with strict patient-level cross-validation, and FMs were used as frozen extractors. MIL models were optimized with Adam (learning rate: 1 × 10^−4^, weight decay: 1 × 10^−5^) for a maximum of 200 epochs, batch size 1. MIL models were implemented using Python 3.10 with PyTorch 2.0.1 (CUDA 11.8). The same hyperparameters were used for all FM-MIL combinations, without combination-specific tuning. The primary performance metric was the concordance index (C-index), which measures the model’s ability to correctly rank patients by mortality risk. The C-index was computed on each held-out fold using Harrell’s estimator and averaged across the 10 folds; fold-level standard deviations are reported in Supplementary Table S12. Time-dependent AUC (tdAUC) results across the 77 pathology FM-MIL combinations and seven ResNet-50 baseline configurations (84 configurations total) at seven timepoints are available in Supplementary Tables S4–S10.

### 2.3. Evaluation of Attention Scores

MIL methods employing attention mechanisms (ABMIL [50], RRTMIL [22], MambaMIL [21]) assign learned weights to patches within a WSI. To assess whether these weights reflect the model’s actual decision-making process, we implemented a perturbation-based faithfulness evaluation following Hense et al. [54]. The procedure involved four steps: (i) Patch Ranking: patches ranked by attention score per WSI; (ii) Progressive Exclusion: highest-attention patches removed in 1% increments; (iii) Performance Tracking: C-index re-evaluated after each exclusion; (iv) Perturbation Curve Generation: C-index plotted as a function of patches dropped. A decline in C-index as the highest-attended patches are removed indicates that the model relies on these regions for its predictions (attention faithfulness). This analysis does not, by itself, establish the biological or histopathological relevance of the attended regions. Control perturbations, i.e., removal of randomly selected or the lowest-attended patches, were not performed in the present study (see Section 4).

### 2.4. Prognostic Analysis

Patients were stratified into high-risk (above median predicted risk score, indicating worse predicted prognosis) and low-risk (below median) groups. To evaluate finer risk gradations, we additionally performed tertile-based analysis (low-, intermediate-, high-risk thirds) and quartile-based analysis (low-, medium-, high-, very high-risk quarters), where lower-numbered groups correspond to lower risk scores and better prognosis. For the full cohort, cut-offs were derived from the pooled held-out risk scores of all 817 patients; for each histologic subgroup, cut-offs were recomputed within that subgroup. To provide survival-appropriate discrimination metrics beyond C-index, we computed time-dependent AUC (tdAUC) at 6, 12, 18, 24, 36, 60, and 120 months for all FM-MIL combinations using the inverse probability of censoring weighting (IPCW) estimator. Clinical variables included: (i) histology (oligodendroglioma, astrocytoma, GBM); (ii) WHO CNS Grade (2, 3, 4); (iii) age at diagnosis, categorized into three groups (18–40, 40–57, 57–89 years) corresponding to the tertiles of the age distribution in the clinical cohort, which yields groups of comparable size (*n* = 220, 217, and 219); (iv) molecular markers: 1p/19q codeletion status and IDH mutation status. Univariate CoxPH analyses assessed the association between predicted risk and OS in the full cohort and within each clinical subgroup. Multivariable CoxPH analyses evaluated whether the risk score remained associated with OS after adjustment for each clinical covariate separately (pairwise models) and for histology, grade, and age simultaneously (fully adjusted model; IDH and 1p/19q status were determined by the WHO 2021 histologic type and were therefore not entered separately). HRs with 95% CIs are reported; statistical significance was assessed using Wald’s test (α = 0.05). The proportional hazards assumption was not formally tested (see Section 4). We distinguish primary from exploratory analyses as follows. The primary analyses were (i) the C-index comparison of all FM-MIL combinations and (ii) the binary Kaplan–Meier and univariate CoxPH analyses of the selected model in the full cohort. All other analyses, including tdAUC, tertile and quartile stratification, subgroup analyses, and multivariable models, are exploratory; their *p*-values are reported without adjustment for multiplicity and should be interpreted as descriptive. For reference, a Bonferroni correction across the nine extreme-group comparisons within the three histologic subgroups (binary, tertile, and quartile stratification) corresponds to a significance threshold of 0.0056.

### 2.5. Embedding Computation and UMAP Visualization

To examine the feature space structure and identify potential technical batch effects, we visualized slide-level embeddings using UMAP [55]. Patch embeddings were aggregated into slide-level vectors via gated-attention pooling [50] using attention parameters trained in the survival MIL model, ensuring that the slide vectors reflect prognostic weighting. UMAP parameters: n_components = 2, n_neighbors = 15, min_dist = 0.1, random_state = 42. To test whether visually distinct clusters reflected staining artefacts, we performed Macenko color deconvolution [56], extracting three hematoxylin (H_0-H_2) and three eosin (E_0-E_2) components. Distributions were compared using the Mann–Whitney U test with Bonferroni correction. Stain normalization and domain adaptation were not applied in the modeling pipeline; FMs were applied to unnormalized patches.

## 3. Results

### 3.1. Benchmarking of FM-MIL Combinations

Table 2 reports cross-validated C-index values for 77 pathology FM-MIL combinations plus seven ResNet-50 baseline configurations (84 configurations total). The highest mean C-index was obtained by UNI2 paired with MambaMIL (0.771), followed closely by H-Optimus-1 + RRTMIL (0.765), Lunit + MeanMIL (0.764), and H-Optimus-1 + MeanMIL (0.762). The leading estimates were UNI2 + MambaMIL 0.771 ± 0.054, H-Optimus-1 + RRTMIL 0.765 ± 0.035, Lunit + MeanMIL 0.764 ± 0.038, and H-Optimus-1 + MeanMIL 0.762 ± 0.046 (mean ± fold-level SD; Supplementary Table S12). The differences in their means were small relative to the observed fold-to-fold variability; we therefore did not consider UNI2 + MambaMIL to be meaningfully superior to these alternatives. The best C-index obtained with 9 out of the 11 domain-specific FMs exceeded the best C-index obtained with the ImageNet-pretrained ResNet-50 baseline (0.732); the exceptions were CONCHv1.5 (0.731) and PRISM (0.720). For each individual MIL method, ResNet-50 was outperformed by at least 8 out of the 11 FMs. DSMIL consistently showed the weakest performance across all FMs (range: 0.553–0.589), only marginally above chance.

To quantify the contribution of each design choice, we decomposed the variability of the C-index values in Table 2 with a two-way analysis of variance using encoder and the MIL method as factors (Supplementary Table S11). Across all seven MIL methods, the MIL method accounted for 93.0% of the total variability and the encoder for 3.8% (both *p* < 0.001), a pattern driven by the consistently lower performance of DSMIL and MaxMIL. Among the remaining five MIL methods, the encoder accounted for 71.1% of the variability and the MIL method for 11.2% (both *p* < 0.001), with 17.8% attributable to the residual, which includes the encoder × MIL interaction. Thus, the choice of aggregation strategy primarily determines whether a combination performs adequately, whereas among adequately performing aggregators, the choice of FM is the larger source of variation. Because one C-index value per combination entered this analysis, the interaction could not be separated from residual error. Spatially aware MIL methods (MambaMIL, RRTMIL, TransMIL) yielded at most modest differences relative to MeanMIL, and no single MIL method ranked first across all FMs, indicating that optimal aggregation is FM-dependent.

Model rankings depended on the performance measure and time horizon (Table 3; Supplementary Tables S4–S10). Although UNI2 + MambaMIL achieved the highest C-index, its tdAUC values were modest (0.603, 0.606, and 0.608 at 12, 24, and 36 months) and ranked between 34th and 69th of the 84 evaluated combinations across the seven timepoints. H-Optimus-1 + RRTMIL achieved higher tdAUC at all three timepoints in Table 3 (0.615–0.654), the ResNet-50 + MambaMIL baseline achieved higher tdAUC at 24 and 36 months (0.642 and 0.653), and GigaPath + TransMIL achieved the highest tdAUC at 24, 36, and 60 months (0.714, 0.702, and 0.687). The C-index summarizes the ranking of all comparable patient pairs over the entire follow-up, whereas tdAUC evaluates, at a fixed horizon, the separation of patients who died before that time from those who survived beyond it, using the inverse probability of censoring weights; the two measures therefore need not agree, particularly under heavy censoring at later timepoints. UNI2 + MambaMIL was selected for the downstream analyses on the basis of the primary metric (C-index) and should be regarded as a representative high-performing combination rather than as uniformly optimal.

### 3.2. Attention Faithfulness Evaluation

**Figure 2:**
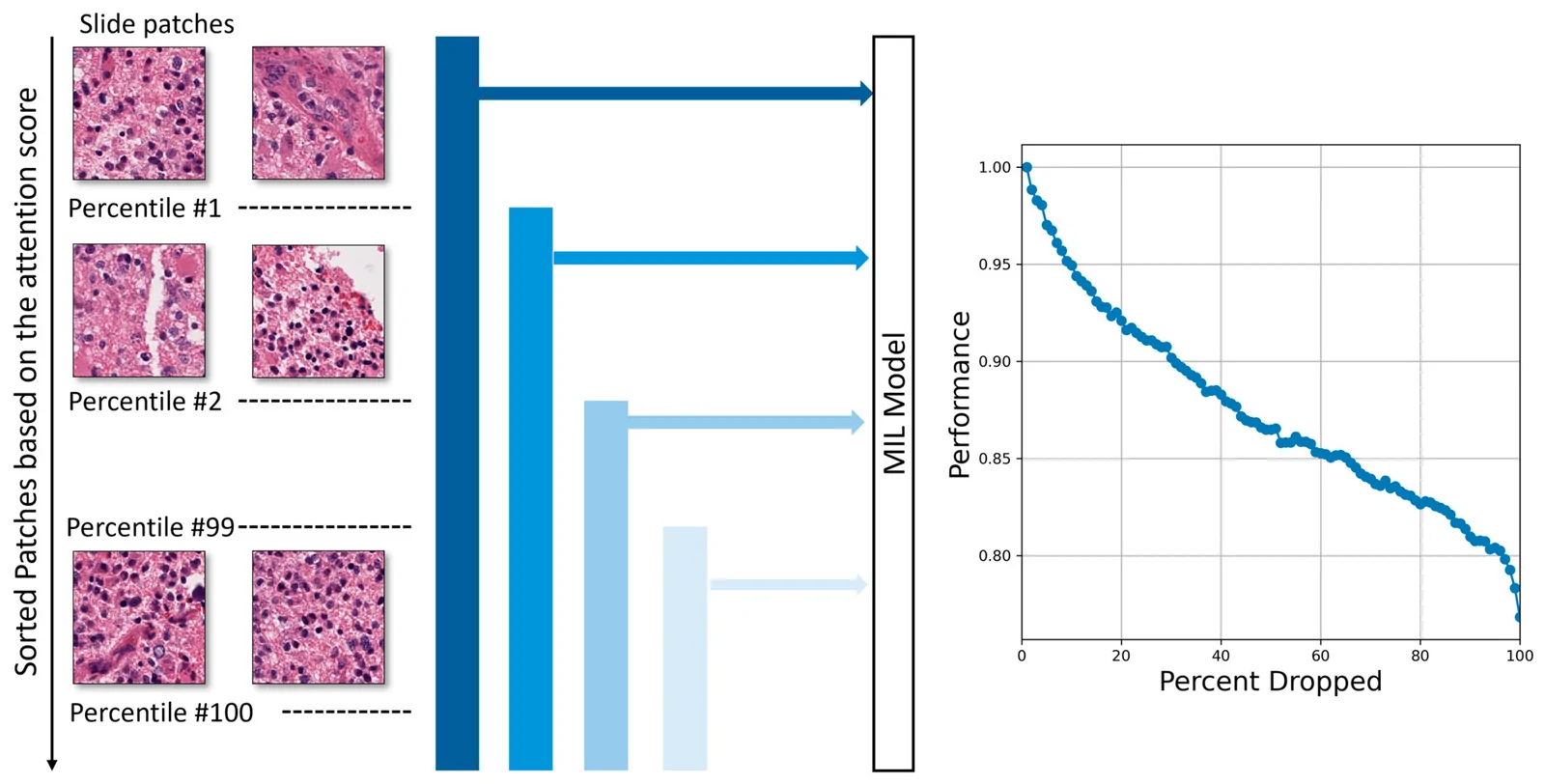
Perturbation-based faithfulness evaluation. Patches are ranked by attention score and progressively removed; performance declines as the highest-attended patches are excluded, indicating that the model relies on the regions it attends to (attention faithfulness). Control perturbations (random or lowest-attention patch removal) were not performed.

Figure 2 presents the perturbation curve for the selected model (UNI2 + MambaMIL). Performance declined progressively as the highest-attended patches were excluded, with the steepest decline when fewer than approximately 20% of patches were removed. This behavior is consistent with the model relying on its highest-attended regions and supports attention faithfulness. However, without control curves obtained by removing random or lowest-attended patches, the extent to which this decline exceeds that expected from removing tissue per se cannot be quantified, and the analysis does not establish that the attended regions are biologically or histopathologically relevant. To our knowledge, this is the first such analysis applied to FM-based survival prediction in ADGs.

### 3.3. Prognostic Stratification

**Figure 3:**
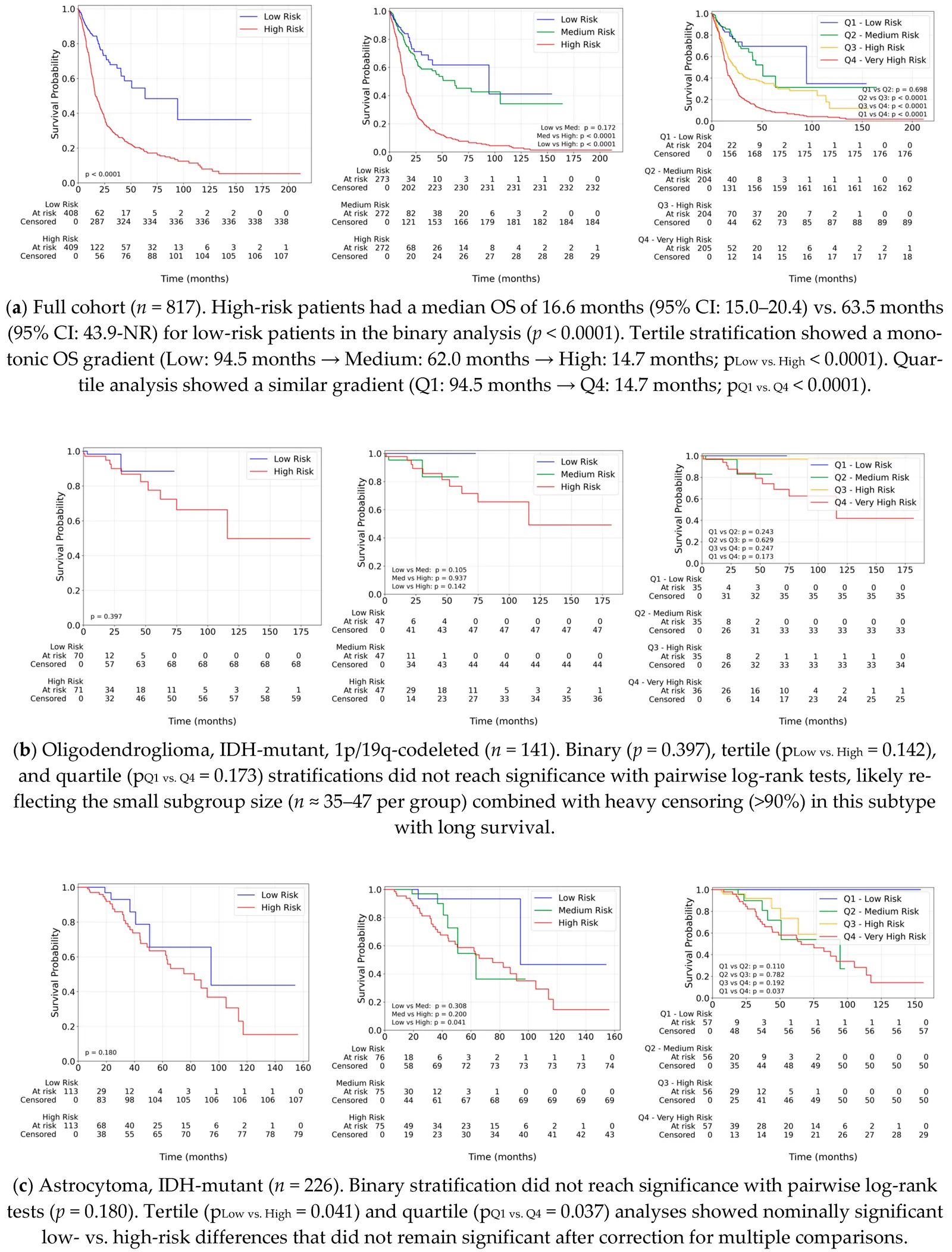

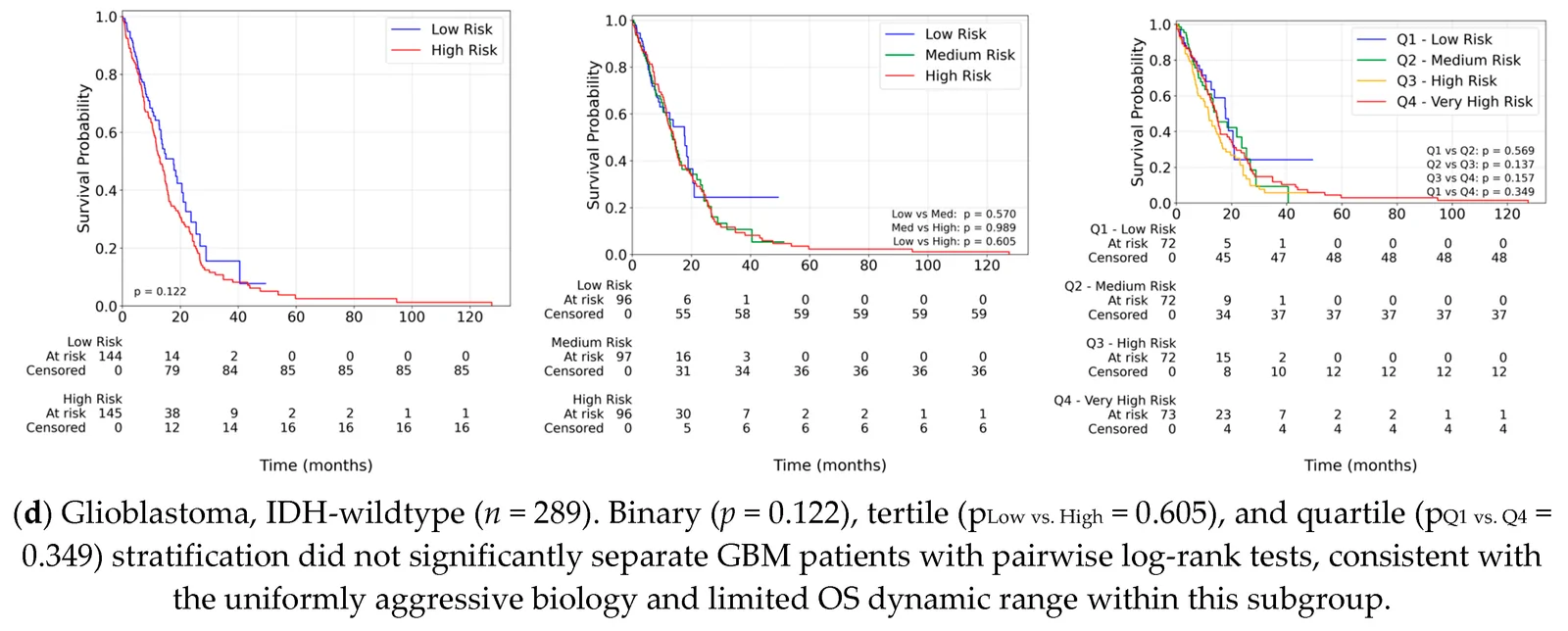
Kaplan–Meier survival curves for model-defined risk groups (UNI2 + MambaMIL) across histologic subgroups. Within each panel, columns show binary median split (**left**), tertile (**middle**), and quartile (**right**) stratification. In panels (**b**–**d**), cut-offs were computed within each histologic subgroup. A higher predicted risk score corresponds to worse prognosis (shorter OS). Numbers at risk and cumulative censored observations are shown below each panel. *p*-values are from log-rank tests and are not adjusted for multiple comparisons. NR = not reached. Detailed median OS with 95% CI for all stratifications are provided in Supplementary Tables S1–S3.

Figure 3 presents the 4 × 3 KM matrix across binary, tertile, and quartile stratifications for the full cohort and each histologic subgroup. In the full cohort (N = 817), high-risk patients had substantially worse OS than low-risk patients: median OS 16.6 months (95% CI: 15.0–20.4) vs. 63.5 months (95% CI: 43.9-NR; log-rank *p* = 9.16 × 10^−17^; HR = 2.90, 95% CI: 2.23–3.76, Table 4). Tertile stratification showed a monotonic gradient in median OS in the full cohort: tertile 1 (lowest risk score): 94.5 months (95% CI: 40.8-NR); tertile 2: 62.0 months (95% CI: 40.5-NR); tertile 3 (highest risk score): 14.7 months (95% CI: 12.4–16.0; p_T1 vs. T3_ = 3.25 × 10^−17^). The two lower tertiles did not differ significantly (p_T1 vs. T2_ = 0.172), indicating that most of the separation occurs between the highest-risk tertile and the remainder. Quartile analysis showed a similar gradient (Q1: 94.5 mo; Q2: 50.8 mo; Q3: 24.1 mo; Q4: 14.7 mo; p_Q1 vs. Q4_ = 1.50 × 10^−12^).

A central finding of this study is that the model did not significantly stratify patients within any individual WHO 2021 type. Binary stratification was not significant in oligodendroglioma (*p* = 0.397), astrocytoma (*p* = 0.180), or GBM (*p* = 0.122). Tertile and quartile stratifications were also not significant within oligodendroglioma (tertile p_T1 vs. T3_ = 0.142; quartile p_Q1 vs. Q4_ = 0.173) or GBM (tertile p_T1 vs. T3_ = 0.605; quartile p_Q1 vs. Q4_ = 0.349). Median OS across GBM tertiles was non-monotonic (17.8, 13.6, and 14.0 months), and oligodendroglioma estimates were based on heavily censored data (>90%), with median OS not reached in most risk groups (Supplementary Tables S2 and S3). Within astrocytoma, the extreme-group comparisons were nominally significant (tertile p_T1 vs. T3_ = 0.041; quartile p_Q1 vs. Q4_ = 0.037) but did not remain significant after Bonferroni correction for the nine within-subgroup extreme-group comparisons (threshold 0.0056) and are therefore hypothesis-generating. Together, these results indicate that a substantial proportion of the discrimination observed in the full cohort reflects the model’s ability to distinguish the major WHO 2021 types, which differ markedly in survival (Supplementary Figure S1), rather than prognostic heterogeneity within each type. Consistent with this interpretation, the risk score was not significantly associated with OS after adjustment for histology in any of the three pairwise histology models (Table 5). Detailed median OS with 95% CIs for all subgroups and stratification levels are provided in Supplementary Tables S1–S3.

In multivariable analyses adjusted for one clinical variable at a time (Table 5, pairwise models), WSI-predicted risk remained significantly associated with OS in 7 out of 11 models: after adjustment for IDH status (HR = 1.44, *p* = 0.033), grade (Grade 4 vs. Grade 2: HR = 1.50, *p* = 0.023; Grade 4 vs. Grade 3: HR = 1.54, *p* = 0.015), 1p/19q status (HR = 2.52, *p* = 4.43 × 10^−8^), and age (all three comparisons). It was not significant after adjustment for histology in any of the three pairwise histology models (*p* = 0.056–0.162) or for Grade 3 vs. Grade 2 (*p* = 0.334). In the fully adjusted model including histology, grade, and age, the risk score was not significantly associated with OS (HR = 1.32, 95% CI: 0.94–1.85, *p* = 0.104). Therefore, independent incremental prognostic value beyond the established clinical and molecular variables was not demonstrated.

### 3.4. Embedding Structure and Batch Effects

**Figure 4:**
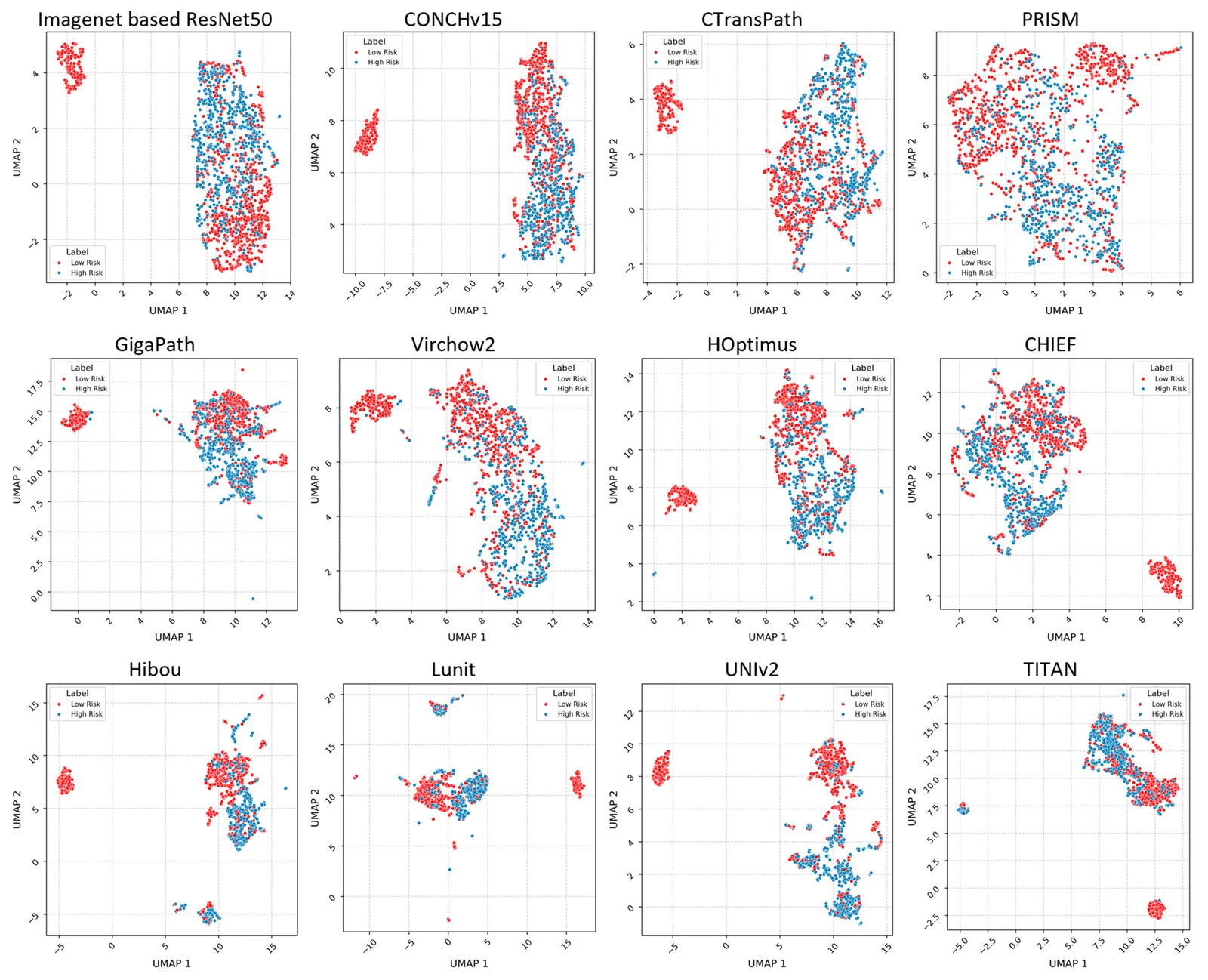
UMAP visualization of feature embeddings from 11 FMs and the ImageNet-pretrained ResNet-50. Colors indicate predicted risk group (median split; high risk = shorter predicted OS).

**Figure 5:**
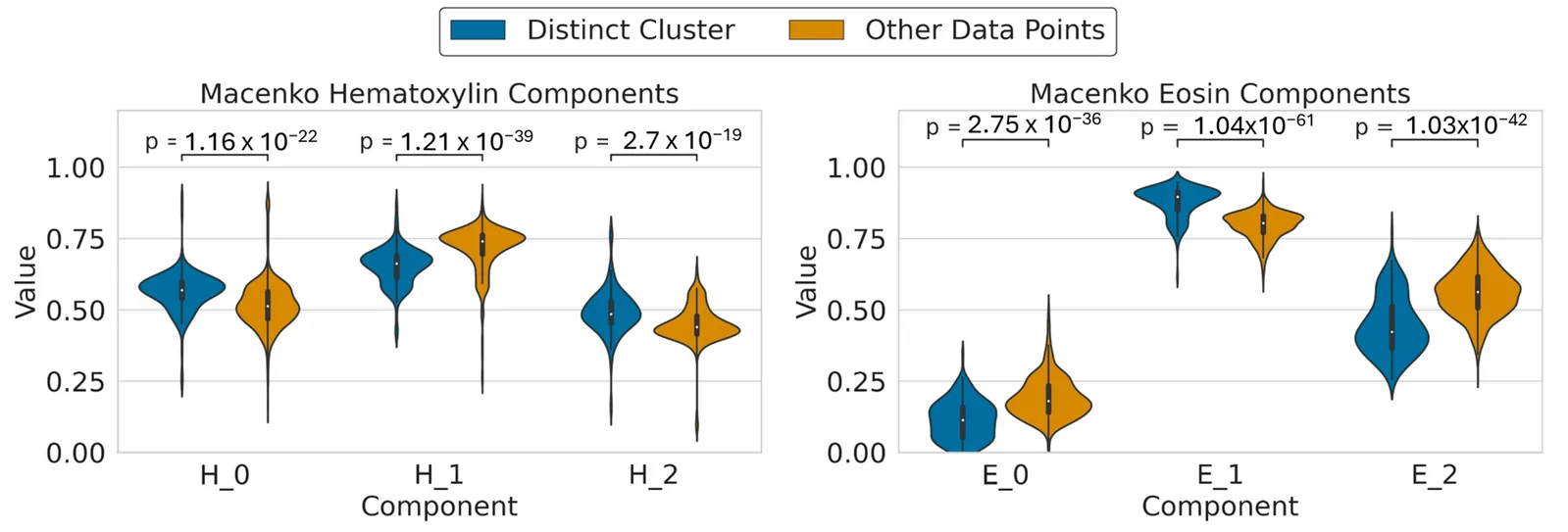
Macenko stain-vector distributions comparing the distinct UMAP cluster (predominantly low-risk slides from a single institution) against all other slides, indicating institutional staining variation as the most likely driver of cluster separation.

Figure 4 and Figure 5 show UMAP projections and Macenko staining analysis. A compact cluster of predominantly low-risk slides (longer predicted survival), consistently observed across all FMs, originated from a single submitting institution. Macenko color deconvolution confirmed highly significant staining differences across all six stain-vector components (all *p* < 10^−18^; H_0: *p* = 1.16 × 10^−22^; H_1: *p* = 1.21 × 10^−39^; H_2: *p* = 2.72 × 10^−19^; E_0: *p* = 2.75 × 10^−36^; E_1: *p* = 1.04 × 10^−61^; E_2: *p* = 1.03 × 10^−42^), indicating institutional staining variation, rather than biology, as the most likely driver of this cluster. Because this cluster was associated with predicted risk, the institution effect is a potential confounder of the reported performance: patients from this institution were present in both training and held-out folds, so the model may partly exploit site-specific staining characteristics. An institution-held-out evaluation was not performed (see Section 4).

## 4. Discussion

To our knowledge, this study presents the first systematic benchmarking of FM-MIL design choices for prognostic risk stratification in ADGs from routine H&E-stained WSIs under the WHO 2021 classification. Through a comprehensive evaluation of 77 pathology FM-MIL combinations together with seven ResNet-50 baseline configurations, the primary contribution is characterizing which design choices drive survival prediction performance, providing guidance for future model development rather than reporting a single optimized model.

An increasing body of literature reports recent advances in analytical data modeling and patch-level analysis based on deep learning showing promise in survival outcomes of ADG patients from H&E slides, both as a classification task and through neural network-based Cox regression models [4]. Notable contributions include multimodal integration of histopathology imaging with molecular profiling to predict OS in glioma patients through the survival convolutional neural networks (SCNNs) ADG [26] and later extended to adult and pediatric cohorts [27]. Despite such advances, most existing approaches rely on ImageNet-pretrained models that capture general-purpose features rather than pathology-specific morphological patterns and tumor heterogeneity. In our benchmark, the best C-index obtained with 9 out of the 11 domain-specific FMs exceeded that of the ImageNet-pretrained ResNet-50 (by up to 0.039, for UNI2), and ResNet-50 was outperformed by at least 8 out of the 11 FMs for every MIL method, consistent with classification task findings [10,11]. The advantage was not uniform: the best results of CONCHv1.5 and PRISM did not exceed the best ResNet-50 result, and ResNet-50 achieved competitive tdAUC at several timepoints. Patch-level, vision-only FMs (UNI2, H-Optimus-1) achieved higher best-case C-index values than slide-level FMs (TITAN, PRISM, CHIEF), although slide-level FMs performed better with MaxMIL. The broader training objective of multimodal models, integrating text alongside image data [57], may dilute fine-grained morphological representations optimized for survival prediction.

Among the MIL methods, spatially aware techniques (MambaMIL, RRTMIL, TransMIL) did not provide consistent improvements over MeanMIL (mean C-index across encoders: 0.742, 0.744, and 0.734, respectively, vs. 0.742 for MeanMIL). MeanMIL achieved competitive performance, suggesting that spatial context relevant to risk stratification is already partially captured within FM embeddings, reducing the incremental value of explicit spatial modeling. DSMIL consistently underperformed across all FMs (C-index: 0.553–0.589), achieving only marginally above chance, and showed near-random time-dependent AUC values (~0.48–0.57) across all evaluated timepoints (Supplementary Tables S4–S10), indicating poor discrimination for this task rather than an inverted predictor. Its dual-stream architecture likely provides redundant signal given the rich representational capacity already encoded by FM embeddings. Notably, MaxMIL showed elevated tdAUC at 6 months (up to 0.86 for certain FM combinations; Supplementary Table S4), while its overall C-index remained lower than attention-based methods. This dissociation suggests that MaxMIL captures morphologic features associated with very early mortality, likely the most aggressive GBM cases, but lacks the discriminative resolution needed for overall survival ranking across the full follow-up period. These findings suggest that for very large, high-capacity FMs, simpler aggregation may be computationally preferable without sacrificing overall prognostic performance.

In the fully adjusted multivariable model, the AI-derived risk score was not significantly associated with OS (HR = 1.32, 95% CI: 0.94–1.85, *p* = 0.104). The present analysis therefore does not demonstrate independent incremental prognostic value of the risk score beyond the established clinical and molecular variables. Possible contributors include correlation between the morphology-derived score and the co-included histology and grade variables, and the limited statistical power of a model with multiple covariates in 656 patients with ~63% censoring; however, these considerations do not change this conclusion. In the pairwise models, the risk score remained significant after adjustment for IDH status, 1p/19q status, age, and two of the three grade contrasts, but not after adjustment for histology, indicating that the prognostic information captured by the score overlaps substantially with the WHO 2021 types.

The absence of significant within-type stratification is a central result of this study. Binary, tertile, and quartile analyses were not significant within oligodendroglioma or GBM, GBM tertiles showed non-monotonic median OS, and oligodendroglioma estimates were limited by heavy censoring; the nominal within-astrocytoma gradients (*p* = 0.041 and *p* = 0.037) did not withstand correction for multiple comparisons. This pattern is consistent with a model trained on a mixed ADG cohort, where the dominant learned signal reflects morphologic correlates of the WHO 2021 types, including IDH mutation status and grade, the dominant prognostic drivers in ADGs. Consequently, the pooled C-index and the separation between risk groups in the full cohort (median OS 16.6 vs. 63.5 months) should be interpreted largely as the ability to recognize these types from H&E morphology rather than as evidence of prognostic resolution within them. A morphology-derived score of this kind could, in principle, provide early prognostic orientation when molecular results are not yet available, for example, to help prioritize molecular testing. Such applications are hypothetical at this stage: the present retrospective analysis did not include external validation, calibration analysis, decision curve analysis, adjustment for treatment, or prospective evaluation, all of which are required before any clinical use. Subtype-specific models trained within each WHO 2021 category may be better suited to address within-class survival heterogeneity.

The perturbation-based faithfulness evaluation introduced here complements static attention heatmaps. The decline in performance upon removal of the highest-attended patches indicates that the model relies on these regions for its predictions, i.e., that attention is faithful to the model. It does not establish whether these regions are biologically or histopathologically relevant, which would require an independent review of high- and low-attention regions by neuropathologists. Moreover, without control curves obtained by random patch removal and by removal of the lowest-attended patches, the specific contribution of the attention ranking cannot be separated from the effect of removing tissue per se. Both analyses are planned for future work, and the perturbation framework should include such controls when used as an interpretability check for attention-based MIL models across computational pathology tasks.

UMAP visualization identified an institution-specific compact cluster that was associated with predicted risk and with significant differences in all six Macenko stain-vector components. We consider this a potential confounder rather than solely a visualization finding. Since slides were randomly assigned to folds using patient-level strata, the model may therefore have partly exploited site-specific staining characteristics, as previously described for TCGA-based deep learning models [58]. The reported cross-validated performance may thus be optimistic with respect to new institutions. Stain normalization and feature-level harmonization were not evaluated experimentally in this study. However, conventional stain normalization should not be assumed to eliminate institution-specific information in pathology foundation-model representations. Recent evaluations have shown that Macenko or Reinhard normalization can reduce site-specific signatures but may leave substantial tissue-source-site information encoded in foundation-model embeddings, while post hoc normalization may only partially mitigate WSI-specific feature clustering [59,60]. Thus, the observed institution-associated embedding cluster may not be fully addressed by conventional stain normalization alone. Dedicated site-preserved validation and the evaluation of stain normalization and feature-level harmonization remain important directions for establishing robustness. Pathology FMs are pretrained on large multi-institutional datasets, which is expected to confer partial robustness to staining variation, yet our results show that institution-specific characteristics remain encoded in their embeddings. A leave-one-institution-out design was not adopted because, in TCGA, submitting institutions differ markedly in the WHO 2021 types, grades, and survival of the patients they contributed, and many contributed few patients or predominantly a single tumor type, since TCGA-LGG and TCGA-GBM were accrued largely from different sets of institutions. Held-out institutions would therefore often lack one or more ADG types or contain too few events, yielding unstable estimates that are not comparable across sites. Future work should evaluate site-preserved cross-validation [58], stain normalization (e.g., Macenko [56]), and feature-level harmonization (e.g., ComBat [61] and fmMAP [62]) experimentally, together with validation in independent cohorts.

This study has several limitations. First, extent of resection and adjuvant treatment data, established prognostic variables in glioma, were not systematically available in TCGA and could not be included in the multivariable models. Future prospective studies with richer clinical annotation should evaluate whether AI-derived risk scores retain independent value after adjusting for treatment-related variables. Second, all performance estimates were internal, patient-level cross-validation estimates; no independent test set or external validation cohort was available. To our knowledge, no other publicly available dataset provides H&E WSIs together with longitudinal OS and the molecular data required for WHO 2021 reclassification across all three ADG types at a comparable scale, and the generalizability of our findings to other institutions, scanners, and staining protocols therefore remains uncertain. Validation on independent, preferably prospectively collected, multi-institutional cohorts is required. Third, the model was trained on a mixed ADG cohort that also included 161 patients without a confident WHO 2021 diagnosis, limiting within-subtype discriminative power; the continuous risk score may better serve as a covariate in future subtype-specific prognostic index development. Fourth, the model used in the downstream analyses was selected as the combination with the highest C-index among the 77 candidates evaluated on the same folds, without nested cross-validation. Selecting the best of many candidates on the same data yields optimistically biased performance estimates [63,64], so the C-index of 0.771 should be regarded as an upper-end estimate; given the small differences among the top-ranked combinations and their fold-level variability, several combinations would be expected to perform comparably on new data. Fifth, the proportional hazards assumption was not formally assessed (e.g., with Schoenfeld residuals [65]); given the markedly different survival distributions of the three ADG types, the reported HRs should be interpreted as averages over follow-up, whereas the C-index, tdAUC, and log-rank analyses do not rely on this assumption. Sixth, age was analyzed as a categorical variable based on cohort tertiles rather than as a continuous variable, which may lose information. Finally, the institution-related staining effect discussed above may have inflated the cross-validated performance estimates.

## 5. Conclusions

Histopathology morphology captured from routine H&E slides encodes prognostic information in adult-type diffuse gliomas, predominantly at the level of the WHO 2021 types. This comprehensive benchmark of 77 pathology FM-MIL combinations plus seven ResNet-50 baseline configurations, is, to our knowledge, the first of this scope under the WHO 2021 criteria, shows that most domain-specific FMs outperform an ImageNet-pretrained encoder, that the MIL aggregation strategy determines whether a combination performs adequately while the FM is the larger source of variation among adequate aggregators, and that model rankings depend on the evaluation metric. Perturbation analysis indicated that the attention mechanisms are faithful to the model’s predictions. In internal cross-validation, the AI-derived risk score separated patients with markedly different OS in the full cohort (median OS 63.5 vs. 16.6 months for low- vs. high-risk patients, and 94.5 vs. 14.7 months for the lowest vs. highest risk tertile), but it did not significantly stratify patients within individual WHO 2021 types and did not show independent prognostic value in the fully adjusted model. The biological relevance of the attended regions, independent incremental prognostic value, robustness to institutional staining variation, generalizability, and clinical utility require further investigation in independent cohorts.

## Figures and Tables

**Table 2:** Cross-validated C-index (mean across the 10 held-out folds) for mortality risk prediction in ADG patients across pretrained models and MIL aggregation methods. A higher predicted risk score corresponds to worse prognosis (shorter OS). Underlined: best MIL per FM. Bold: best FM per MIL. Square brackets: overall best combination. Fold-level standard deviations are provided in Supplementary Table S12.

FM	MeanMIL	MaxMIL	ABMIL	DSMIL	TransMIL	RRTMIL	MambaMIL
ResNet-50	0.718	0.672	0.692	0.553	0.710	0.731	0.732
Lunit	**0.764**	**0.715**	0.742	0.563	0.758	0.762	0.753
GigaPath	0.748	0.655	**0.750**	0.576	0.732	0.754	0.750
CTransPath	0.739	0.679	0.738	0.560	0.736	0.743	0.736
Hibou	0.750	0.697	0.744	0.585	0.722	0.757	0.758
H-Optimus-1	0.762	0.696	0.744	**0.589**	**0.760**	**0.765**	0.748
Virchow2	0.752	0.686	0.729	0.559	0.735	0.753	0.729
CONCHv1.5	0.731	0.680	0.702	0.582	0.711	0.720	0.725
UNI2	0.759	0.672	0.748	0.575	0.738	0.760	**[0.771]**
CHIEF	0.738	0.713	0.733	0.569	0.750	0.742	0.750
PRISM	0.716	0.688	0.702	0.573	0.720	0.715	0.715
TITAN	0.725	0.709	0.719	0.565	0.740	0.731	0.734

**Table 3:** Time-dependent AUC (tdAUC) at 12, 24, and 36 months for the three combinations with the highest C-index and the ResNet-50 baseline, computed using IPCW. Full results across all 84 encoder-MIL configurations and seven timepoints are provided in Supplementary Tables S4–S10.

FM-MIL	tdAUC @ 12 mo	tdAUC @ 24 mo	tdAUC @ 36 mo
UNI2 + MambaMIL	0.603	0.606	0.608
H-Optimus-1 + RRTMIL	0.615	0.631	0.654
Lunit + MeanMIL	0.547	0.583	0.602
ResNet-50 + MambaMIL (baseline)	0.601	0.642	0.653

**Table 4:** Univariate CoxPH models evaluating the association between model-predicted risk (high vs. low) and OS. High risk = predicted risk score above the median of the respective cohort or subgroup (worse prognosis). The “Clinical” row was restricted to the 656 patients with a confident WHO 2021 diagnosis. Oligo, oligodendroglioma; Astro, astrocytoma. * *p* < 0.05 (Wald’s test).

Group	Cohort	N	Censored	HR	95% CI	*p*-Value
	All patients	817	446	2.90	2.23–3.76	1.84 × 10^−15^ *
	Clinical	656	415	2.79	2.01–3.87	8.28 × 10^−10^ *
Histology	Oligo	141	128	1.97	0.41–9.54	3.97 × 10^−1^
	Astro	226	186	1.82	0.76–4.38	1.80 × 10^−1^
	GBM	289	101	1.28	0.94–1.76	1.22 × 10^−1^
Grade	Grade 2	181	166	1.69	0.36–7.98	5.05 × 10^−1^
	Grade 3	155	133	1.59	0.46–5.52	4.66 × 10^−1^
	Grade 4	318	114	1.42	1.05–1.92	2.17 × 10^−2^ *
IDH	IDH-wildtype	289	101	1.28	0.94–1.76	1.22 × 10^−1^
	IDH-mutant	367	314	1.65	0.76–3.55	2.03 × 10^−1^
1p/19q	Non-codel	511	284	2.40	1.75–3.29	4.94 × 10^−8^ *
	Co-deleted	141	128	1.97	0.41–9.54	3.97 × 10^−1^
Age	18–40 years	220	184	2.77	0.97–7.88	5.69 × 10^−2^
	40–57 years	217	145	2.31	1.25–4.28	7.75 × 10^−3^ *
	57–89 years	219	86	1.52	1.06–2.20	2.43 × 10^−2^ *

**Table 5:** Multivariable CoxPH models assessing WSI-predicted risk alongside individual (pairwise models) and combined (fully adjusted model) clinical covariates. Reference categories are shown in braces. Oligo, oligodendroglioma; Astro, astrocytoma; G, WHO CNS grade. * *p* < 0.05 (Wald’s test).

Model/Covariates {Reference}	N	Censored	HR	95% CI	*p*
**Risk + Histology (Astro vs. Oligo)**	367	314			
Risk {low}			1.95	0.98–3.87	0.056
Astro {Oligo}			1.72	0.92–3.23	0.090
**Risk + Histology (GBM vs. Oligo)**	430	229			
Risk {low}			1.30	0.90–1.88	0.162
GBM {Oligo}			7.82	5.37–11.40	1.06 × 10^−26^ *
**Risk + Histology (GBM vs. Astro)**	515	287			
Risk {low}			1.39	0.98–1.96	0.066
GBM {Astro}			3.57	2.66–4.80	2.92 × 10^−17^ *
**Risk + Grade (G3 vs. G2)**	336	299			
Risk {low}			1.47	0.67–3.23	0.334
G3 {G2}			2.11	1.10–4.10	0.027 *
**Risk + Grade (G4 vs. G2)**	499	280			
Risk {low}			1.50	1.06–2.13	0.023 *
G4 {G2}			3.72	2.83–4.90	4.01 × 10^−21^ *
**Risk + Grade (G4 vs. G3)**	473	247			
Risk {low}			1.54	1.09–2.17	0.015 *
G4 {G3}			6.96	4.40–11.01	1.10 × 10^−16^ *
**Risk + IDH**	656	415			
Risk {low}			1.44	1.03–2.02	0.033 *
IDH-wt {IDH-mut}			9.14	6.48–12.90	2.32 × 10^−36^ *
**Risk + 1p/19q**	652	412			
Risk {low}			2.52	1.81–3.50	4.43 × 10^−8^ *
Non-codel {codel}			4.96	2.82–8.73	2.74 × 10^−8^ *
**Risk + Age (40–57 vs. 18–40)**	437	329			
Risk {low}			3.06	1.82–5.14	2.41 × 10^−5^ *
Age 40–57 {18–40}			2.40	1.60–3.60	2.62 × 10^−5^ *
**Risk + Age (57–89 vs. 18–40)**	439	270			
Risk {low}			1.79	1.21–2.64	0.004 *
Age 57–89 {18–40}			2.50	2.06–3.04	4.37 × 10^−20^ *
**Risk + Age (57–89 vs. 40–57)**	436	231			
Risk {low}			1.90	1.34–2.71	0.0004 *
Age 57–89 {40–57}			2.30	1.71–3.09	3.61 × 10^−8^ *
**Risk + All Clinical Covariates (fully adjusted)**	656	415			
Risk {low}			1.32	0.94–1.85	0.104
Astro {Oligo}			1.58	0.79–3.16	0.197
GBM {Oligo}			2.82	1.19–6.71	0.019 *
G3 {G2}			2.01	1.03–3.90	0.040 *
G4 {G2}			6.30	3.02–13.14	9.30 × 10^−7^ *
Age 40–57 {18–40}			1.47	0.93–2.32	0.097
Age 57–89 {40–57}			2.68	1.71–4.21	1.77 × 10^−5^ *

## Data Availability

The TCGA datasets are publicly available via the Genomic Data Commons portal at https://portal.gdc.cancer.gov/.

## Supplementary Materials

The following supporting information can be downloaded at: https://www.mdpi.com/article/10.3390/cancers18203259/s1, Figure S1: Kaplan–Meier survival curves stratified by clinical and molecular features; Figure S2: Patient selection flowchart; Table S1: Median OS by binary risk group; Table S2: Median OS by tertile risk group; Table S3: Median OS by quartile risk group; Tables S4–S10: Time-dependent AUC at 6, 12, 18, 24, 36, 60, and 120 months; Table S11: Two-way analysis of variance of the C-index values in Table 2; Table S12: Fold-level variability of the cross-validated C-index.

## Abbreviations

Abbreviation	Definition
ADG	Adult-type diffuse glioma
AI	Artificial intelligence
C-index	Concordance index
CI	Confidence interval
CNS	Central nervous system
CoxPH	Cox proportional hazards
FFPE	Formalin-fixed paraffin-embedded
FM	Foundation model
GBM	Glioblastoma
H&E	Hematoxylin and eosin
HR	Hazard ratio
IDH	Isocitrate dehydrogenase
IPCW	Inverse probability of censoring weighting
KM	Kaplan–Meier
MIL	Multiple instance learning
NLL	Negative log-likelihood
OS	Overall survival
SD	Standard deviation
TCGA	The Cancer Genome Atlas
tdAUC	Time-dependent area under the curve
UMAP	Uniform Manifold Approximation and Projection
WHO	World Health Organization
WSI	Whole-slide image
