# Supplementary material for: AI-Based Prognostic Risk Stratification of Adult-Type Diffuse Glioma Patients from H&E-Stained Slides
Source: Cancers (Basel). 2026 Oct 9;18(20):3259. doi: 10.3390/cancers18203259 (PMC13702140; doi:10.3390/cancers18203259)
Supplement: Supplementary file 1 [file cancers-18-03259-s001.zip › cancers-4574018-supplementary.pdf]

Supplementary Materials

AI-based prognostic risk stratification of adult-type diffuse glioma patients from H&E-stained slides

Shubham Innani, Carla Pitarch-Abaigar, Marwan M. Majeed, Hannah Harmsen, W. Robert Bell, Dimitrios Makris, and Spyridon Bakas*

** Correspondence: spbakas@iu.edu*

S1. Median OS by Binary Risk Group (Median Split)

Patients were divided into high-risk (predicted risk score above the median of the respective cohort or subgroup, indicating worse predicted prognosis and shorter OS) and low-risk (below median, better predicted prognosis and longer OS) groups. Risk scores were derived from the combination with the highest C-index (UNI2 + MambaMIL). For histologic subgroups, cut-offs were computed within each subgroup.

Table S1. Median OS (months, 95% CI) by binary risk group for the full cohort and key histologic subgroups. High risk = predicted risk score above the median of the respective cohort or subgroup (shorter predicted OS). NR = not reached. † Binary stratification does not significantly separate GBM (p = 0.122); see Table S2 for tertile analysis within GBM.

Subgroup	N	Median OS, High Risk (mo)	Median OS, Low Risk (mo)	Log-rank p
Full cohort	817	16.6 (15.0-20.4)	63.5 (43.9-NR)	9.16×10^{-17}
Oligodendroglioma	141	115.8 (75.0-NR)	NR (NR-NR)	0.397
Astrocytoma	226	82.5 (50.5-105.1)	94.5 (50.8-NR)	0.173
Glioblastoma†	289	13.0 (11.2-15.2)	17.7 (13.3-21.0)	0.122

S2. Median OS by Tertile Risk Group

Patients were divided into three equal groups based on their continuous predicted risk score, with cut-offs computed within the full cohort or within each histologic subgroup. Tertile 1 (T1) = lowest risk scores (best predicted prognosis, longest OS); Tertile 3 (T3) = highest risk scores (worst predicted prognosis, shortest OS).

Table S2. Median OS (months, 95% CI) by tertile risk group. T1 = lowest risk score (best prognosis, longest OS); T3 = highest risk score (worst prognosis, shortest OS). NR = not reached.

Subgroup	N	OS T1 (mo)	OS T2 (mo)	OS T3 (mo)	p T1-T2	p T2-T3	p T1-T3
Full cohort	817	94.5 (40.8-NR)	62.0 (40.5-NR)	14.7 (12.4-16.0)	0.172	2.45×10^{-21}	3.25×10^{-17}
Oligodendroglioma	141	NR (NR-NR)	NR (30.2-NR)	115.8 (62.0-NR)	0.105	0.937	0.142
Astrocytoma	226	94.5 (94.5-NR)	63.5 (43.9-NR)	75.1 (44.4-105.1) (44.4- 105.1)	0.308	0.200	0.041
Glioblastoma	289	17.8 (10.7-20.5)	13.6 (10.4-16.6) (10.4-16.6)	14.0 (11.5-15.9)	0.570	0.989	0.605

Note on oligodendroglioma tertile: The non-monotonic OS pattern in oligodendroglioma tertiles (T1: NR; T2: NR; T3: 115.8 mo) reflects the very small subgroup size (N ≈ 47 per tertile) combined with heavy censoring (91%), making Kaplan-Meier estimates unreliable. None of the pairwise comparisons reached significance (p = 0.142 for T1 vs. T3).

Note on GBM tertile: The non-monotonic GBM tertile pattern (T1: 17.8 mo; T2: 13.6 mo; T3: 14.0 mo) with no significant pairwise comparisons (p = 0.605 for T1 vs. T3) indicates that the risk score does not resolve survival differences within GBM. Tertile cut-offs were computed within the GBM subgroup. GBM-specific models trained within this subtype may better characterize within-GBM heterogeneity.

S3. Median OS by Quartile Risk Group

Patients were divided into four equal groups, with cut-offs computed within the full cohort or within each histologic subgroup. Q1 = lowest predicted risk scores (best predicted prognosis); Q4 = highest predicted risk scores (worst predicted prognosis).

Table S3. Median OS (months, 95% CI) by quartile risk group. Q1 = lowest risk score (best prognosis, longest OS); Q4 = highest risk score (worst prognosis, shortest OS). NR = not reached. † Estimates for oligodendroglioma and GBM should be interpreted with caution due to small subgroup size (N ≈ 35-73 per quartile) and heavy censoring.

Subgroup	N	OS Q1 (mo)	OS Q2 (mo)	OS Q3 (mo)	OS Q4 (mo)	p Q1-Q2	p Q1-Q4
Full cohort	817	94.5 (94.5-NR)	50.8 (40.5-NR)	24.1 (16.8-31.6)	14.7 (12.6-16.0)	3.91×10^{-5}	1.50×10^{-12}
Oligodendroglioma†	141	NR (NR-NR)	NR (30.2-NR)	62.0 (52.1-NR)	115.8 (62.0-NR)	3.67×10^{-6}	0.173
Astrocytoma	226	NR (62.1-NR)	94.5 (43.9-NR)	94.5 (50.5-NR)	65.7 (38.7-105.1) 38.7-105.1)	0.110	0.037
Glioblastoma†	289	17.8 (12.6-21.0) (12.6-21.0)	14.6 (10.3-23.7)	11.7 (7.6-14.7)	14.7 (11.2-16.0)	0.086	0.349

The full cohort shows a monotonic decrease in median OS from Q1 (94.5 mo) to Q4 (14.7 mo; $p = 1.50 \times 10^{-12}$ for Q1 vs. Q4). Within histologic subgroups, only the astrocytoma Q1 vs. Q4 comparison was nominally significant (p = 0.037), and it does not remain significant after correction for multiple comparisons.

S4. Kaplan-Meier Survival Curves Stratified by Molecular and Clinical Features

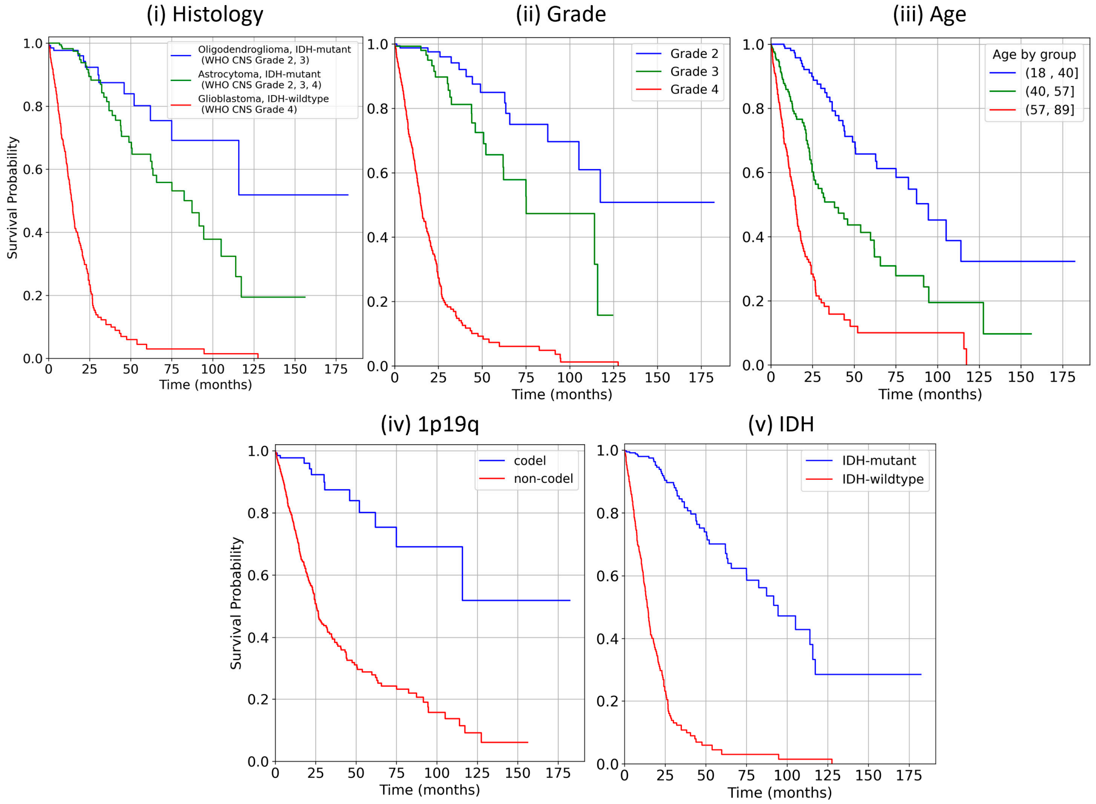

Figure S1. Kaplan-Meier survival curves for different prognostic markers in the overall cohort of adult-type diffuse glioma (ADG) patients. The curves are stratified by (i) histology, (ii) WHO grade, (iii) age at diagnosis, (iv) 1p/19q codeletion status, and (v) IDH status, illustrating survival probabilities over time for each subgroup.

Overall survival differed markedly across subgroups. Patients with oligodendroglioma (IDH-mutant) and lower-grade tumors (Grade 2) showed the most favorable outcomes, while glioblastoma (IDH-wildtype) and Grade 4 tumors exhibited the poorest survival. Younger patients demonstrated improved survival compared to older age groups. The presence of 1p/19q co-deletion was associated with significantly better prognosis relative to non-codeleted tumors. Similarly, IDH-mutant status conferred a substantial survival advantage compared to IDH-wildtype tumors. Collectively, these results highlight the strong prognostic impact of histology, tumor grade, age, and key molecular markers.

S5. Time-Dependent AUC at Multiple Timepoints

Time-dependent AUC (tdAUC) was computed using the inverse probability of censoring weighting (IPCW) estimator at 6, 12, 18, 24, 36, 60, and 120 months for all 77 pathology FM-MIL combinations and the seven ResNet-50 baseline configurations (84 configurations total) (full cohort, N = 817). A value > 0.5 indicates better-than-random discrimination at that timepoint. Three noteworthy patterns are observed across these tables. First, DSMIL shows consistently near-random discrimination (~0.48-0.57) across all timepoints, consistent with its low C-index (0.553-0.589), indicating poor discrimination for this task. Second, MaxMIL shows elevated tdAUC at 6 months (up to 0.861 for CONCHv1.5; Table S4) that converge toward other methods at longer timepoints, suggesting MaxMIL captures morphologic features associated with very early mortality rather than providing overall prognostic discrimination. Third, rankings by tdAUC differ from rankings by C-index: UNI2 + MambaMIL, the combination with the highest C-index, showed modest tdAUC values (0.588-0.638) and ranked between 34th and 69th of the 84 combinations across timepoints.

Table S4. Time-dependent AUC at 6 months.

Encoder	ABMIL	DSMIL	MambaMIL	MaxMIL	MeanMIL	RRTMIL	TransMIL
CHIEF	0.537	0.582	0.485	0.618	0.562	0.473	0.661
CONCHv1.5	0.728	0.379	0.551	0.861	0.667	0.570	0.600
CTransPath	0.436	0.559	0.526	0.755	0.456	0.406	0.501
GigaPath	0.593	0.628	0.428	0.797	0.608	0.608	0.664
Hibou	0.553	0.476	0.528	0.715	0.615	0.475	0.533
H-Optimus-1	0.622	0.576	0.557	0.767	0.663	0.512	0.573
ResNet-50	0.552	0.485	0.488	0.728	0.687	0.658	0.614
Lunit	0.534	0.411	0.556	0.730	0.462	0.590	0.606
PRISM	0.413	0.381	0.601	0.481	0.431	0.498	0.618
TITAN	0.575	0.564	0.512	0.670	0.589	0.609	0.630
UNI2	0.479	0.638	0.595	0.720	0.565	0.533	0.650
Virchow2	0.591	0.608	0.547	0.761	0.498	0.610	0.717

Table S5. Time-dependent AUC at 12 months.

Encoder	ABMIL	DSMIL	MambaMIL	MaxMIL	MeanMIL	RRTMIL	TransMIL
CHIEF	0.586	0.520	0.611	0.646	0.599	0.592	0.570
CONCHv1.5	0.645	0.554	0.566	0.760	0.596	0.606	0.665
CTransPath	0.567	0.593	0.609	0.676	0.604	0.569	0.585
GigaPath	0.590	0.543	0.598	0.682	0.590	0.550	0.704
Hibou	0.593	0.437	0.559	0.630	0.626	0.573	0.531
H-Optimus-1	0.657	0.595	0.616	0.683	0.633	0.615	0.626
ResNet-50	0.670	0.452	0.601	0.672	0.654	0.594	0.606
Lunit	0.623	0.434	0.577	0.675	0.547	0.513	0.592
PRISM	0.445	0.404	0.606	0.520	0.518	0.524	0.597
TITAN	0.571	0.501	0.591	0.631	0.631	0.611	0.557
UNI2	0.525	0.555	0.603	0.686	0.589	0.577	0.630
Virchow2	0.535	0.555	0.593	0.656	0.541	0.600	0.711

Table S6. Time-dependent AUC at 18 months.

Encoder	ABMIL	DSMIL	MambaMIL	MaxMIL	MeanMIL	RRTMIL	TransMIL
CHIEF	0.641	0.538	0.646	0.673	0.651	0.639	0.609
CONCHv1.5	0.664	0.536	0.633	0.749	0.649	0.634	0.698
CTransPath	0.594	0.562	0.657	0.698	0.634	0.621	0.652
GigaPath	0.628	0.527	0.637	0.669	0.642	0.583	0.709
Hibou	0.655	0.474	0.633	0.661	0.659	0.635	0.619
H-Optimus-1	0.661	0.524	0.651	0.674	0.653	0.630	0.631
ResNet-50	0.693	0.449	0.673	0.713	0.689	0.650	0.646
Lunit	0.636	0.394	0.613	0.690	0.590	0.566	0.633
PRISM	0.541	0.456	0.638	0.612	0.603	0.599	0.654
TITAN	0.581	0.482	0.594	0.649	0.644	0.624	0.595
UNI2	0.588	0.554	0.638	0.680	0.635	0.618	0.646
Virchow2	0.588	0.529	0.591	0.649	0.586	0.628	0.725

Table S7. Time-dependent AUC at 24 months.

Encoder	ABMIL	DSMIL	MambaMIL	MaxMIL	MeanMIL	RRTMIL	TransMIL
CHIEF	0.656	0.546	0.652	0.681	0.665	0.664	0.631
CONCHv1.5	0.645	0.523	0.615	0.713	0.617	0.617	0.661
CTransPath	0.575	0.546	0.640	0.664	0.627	0.615	0.636
GigaPath	0.655	0.534	0.622	0.678	0.650	0.591	0.714
Hibou	0.651	0.506	0.637	0.626	0.654	0.628	0.616
H-Optimus-1	0.664	0.496	0.616	0.669	0.622	0.631	0.599

ResNet-50	0.675	0.472	0.642	0.680	0.663	0.614	0.587
Lunit	0.604	0.412	0.625	0.670	0.583	0.550	0.602
PRISM	0.541	0.459	0.670	0.635	0.606	0.571	0.619
TITAN	0.582	0.498	0.584	0.634	0.634	0.612	0.565
UNI2	0.607	0.539	0.606	0.663	0.617	0.592	0.611
Virchow2	0.542	0.548	0.574	0.632	0.604	0.610	0.680

Table S8. Time-dependent AUC at 36 months.

Encoder	ABMIL	DSMIL	MambaMIL	MaxMIL	MeanMIL	RRTMIL	TransMIL
CHIEF	0.644	0.567	0.664	0.665	0.655	0.677	0.634
CONCHv1.5	0.661	0.518	0.633	0.701	0.644	0.630	0.649
CTransPath	0.606	0.539	0.660	0.639	0.634	0.657	0.648
GigaPath	0.638	0.513	0.639	0.647	0.645	0.605	0.702
Hibou	0.660	0.505	0.650	0.637	0.651	0.629	0.616
H-Optimus-1	0.667	0.505	0.633	0.654	0.621	0.654	0.611
ResNet-50	0.697	0.498	0.653	0.685	0.662	0.625	0.598
Lunit	0.622	0.450	0.663	0.640	0.602	0.571	0.628
PRISM	0.574	0.486	0.640	0.636	0.613	0.606	0.639
TITAN	0.587	0.504	0.594	0.621	0.626	0.608	0.557
UNI2	0.577	0.494	0.608	0.637	0.608	0.603	0.628
Virchow2	0.584	0.571	0.576	0.625	0.613	0.607	0.671

Table S9. Time-dependent AUC at 60 months.

Encoder	ABMIL	DSMIL	MambaMIL	MaxMIL	MeanMIL	RRTMIL	TransMIL
CHIEF	0.654	0.487	0.646	0.673	0.663	0.665	0.654
CONCHv1.5	0.658	0.536	0.589	0.661	0.631	0.604	0.600
CTransPath	0.631	0.513	0.646	0.638	0.664	0.623	0.620
GigaPath	0.626	0.483	0.639	0.633	0.641	0.614	0.687
Hibou	0.631	0.486	0.609	0.644	0.648	0.634	0.624
H-Optimus-1	0.634	0.467	0.627	0.637	0.629	0.648	0.603
ResNet-50	0.660	0.503	0.625	0.668	0.652	0.634	0.620
Lunit	0.608	0.505	0.642	0.651	0.643	0.602	0.611
PRISM	0.603	0.490	0.630	0.611	0.610	0.612	0.644
TITAN	0.588	0.479	0.595	0.606	0.635	0.619	0.560
UNI2	0.600	0.478	0.588	0.613	0.605	0.611	0.603
Virchow2	0.603	0.544	0.559	0.618	0.627	0.609	0.663

Table S10. Time-dependent AUC at 120 months.

Encoder	ABMIL	DSMIL	MambaMIL	MaxMIL	MeanMIL	RRTMIL	TransMIL
CHIEF	0.637	0.483	0.617	0.641	0.632	0.638	0.594
CONCHv1.5	0.622	0.484	0.595	0.649	0.601	0.592	0.579
CTransPath	0.593	0.501	0.590	0.616	0.635	0.599	0.618
GigaPath	0.619	0.497	0.631	0.625	0.629	0.613	0.653
Hibou	0.623	0.477	0.583	0.622	0.612	0.607	0.604
H-Optimus-1	0.617	0.478	0.598	0.646	0.603	0.620	0.605
ResNet-50	0.660	0.505	0.594	0.651	0.647	0.604	0.633
Lunit	0.602	0.540	0.615	0.647	0.625	0.602	0.611
PRISM	0.618	0.475	0.610	0.627	0.606	0.601	0.609
TITAN	0.618	0.493	0.574	0.596	0.626	0.590	0.578
UNI2	0.598	0.487	0.598	0.605	0.601	0.607	0.593
Virchow2	0.627	0.505	0.595	0.618	0.619	0.610	0.624

S6. Patient Selection

Figure S2 summarizes the cohort. All 817 subjects with diagnostic WSIs and OS data were used for model development and pooled cross-validated evaluation, because the training target (OS) does not depend on the diagnostic label. Of these, 656 subjects (1,322 WSIs) were confidently reclassified under WHO 2021 criteria and form the clinical cohort for subgroup, CoxPH, and multivariable analyses. The remaining 161 subjects (306 WSIs) had valid OS data, but the available molecular information was insufficient, or the molecular profile could not be mapped unambiguously to one of the three ADG types; they were excluded from all analyses that require a WHO 2021 diagnosis.

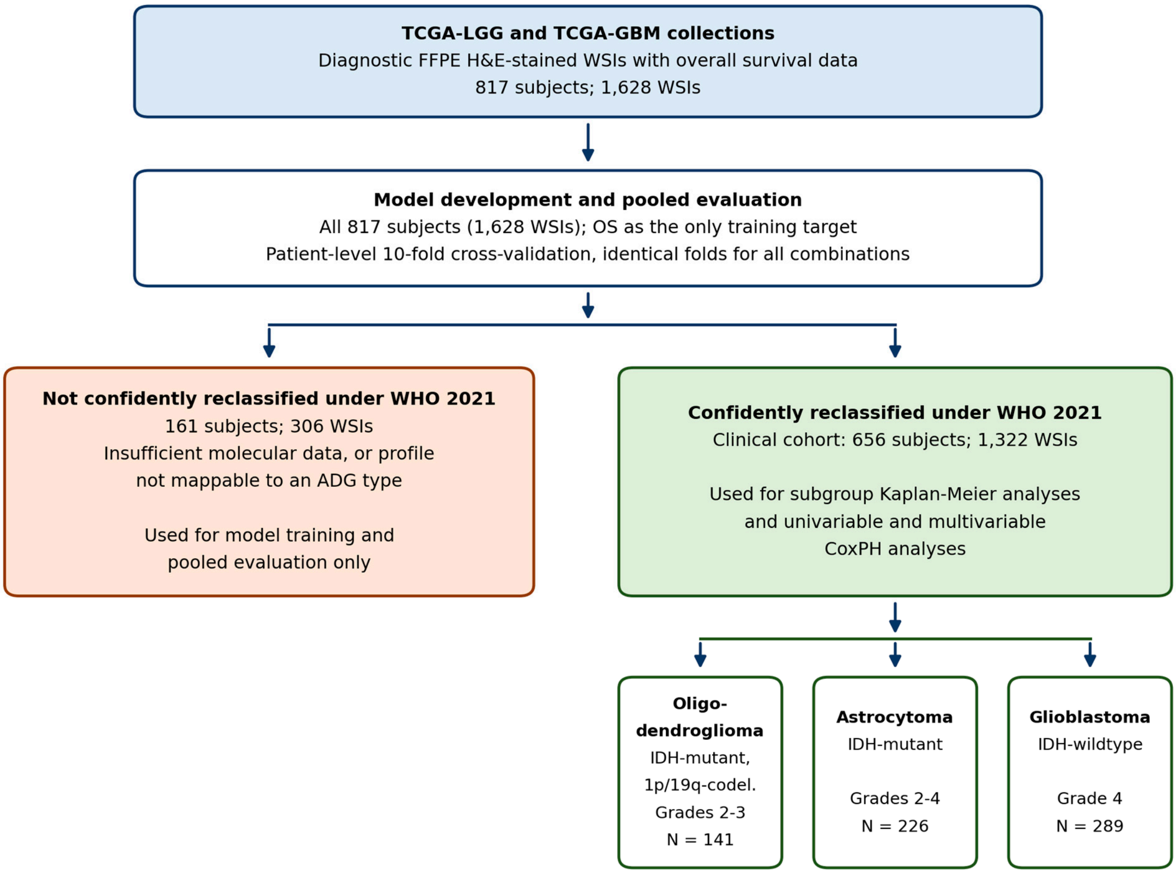

Figure S2. Patient selection flowchart. The pooled cross-validated performance estimates are based on all 817 subjects; all analyses that require a WHO 2021 diagnosis are restricted to the 656 confidently reclassified subjects.

S7. Variance Decomposition of C-index Values

To quantify the contribution of the encoder and of the MIL method to performance, the 84 C-index values in Table 2 of the main manuscript (12 encoders, including the ResNet-50 baseline, × 7 MIL methods) were analyzed with a two-way analysis of variance without replication. Because a single value per combination was available, the encoder x MIL interaction cannot be separated from residual error and is contained in the residual term. The analysis was repeated after excluding DSMIL and MaxMIL, which performed consistently worse than the other MIL

methods. When all MIL methods are included, the MIL method dominates the variability; among the five adequately performing MIL methods, the encoder is the larger source of variation.

Table S11. Two-way analysis of variance of the cross-validated C-index values in main Table 2. SS, sum of squares; df, degrees of freedom.

Analysis set	Source of variation	% of total SS	df	F	p
All MIL methods (12 × 7)	Encoder	3.8	11	7.15	< 0.001
	MIL method	93.0	6	322.43	< 0.001
	Residual (incl. encoder × MIL)	3.2	66		
Excluding DSMIL and MaxMIL (12 × 5)	Encoder	71.1	11	16.01	< 0.001
	MIL method	11.2	4	6.91	< 0.001
	Residual (incl. encoder × MIL)	17.8	44		

S8. Fold-Level Variability of the Cross-Validated C-index

Table 2 of the main manuscript reports, for each encoder-MIL configuration, the mean C-index across the 10 held-out folds of the patient-level cross-validation. Table S12 reports the corresponding fold-to-fold standard deviation where the fold-level run summary was available for this revision. The same fold assignment was used across configurations. The leading combinations show substantial fold-to-fold variability relative to the small differences in their mean C-index values (UNI2 + MambaMIL: 0.771 ± 0.054; H-Optimus-1 + RRTMIL: 0.765 ± 0.035; Lunit + MeanMIL: 0.764 ± 0.038; H-Optimus-1 + MeanMIL: 0.762 ± 0.046), and no single combination should be regarded as meaningfully superior to its closest alternatives. The Virchow2 fold-level SD values remain to be inserted from the corresponding run summary.

Table S12. Cross-validated C-index for all encoder-MIL configurations, reported as mean ± standard deviation across the 10 held-out folds where available. Virchow2 SD entries are retained as placeholders pending insertion from the fold-level run summary.

FM	MeanMIL	MaxMIL	ABMIL	DSMIL	TransMIL	RRTMIL	MambaMIL
ResNet-50	0.718 ± 0.046	0.672 ± 0.041	0.692 ± 0.044	0.553 ± 0.027	0.710 ± 0.037	0.731 ± 0.046	0.732 ± 0.036
Lunit	0.764 ± 0.038	0.715 ± 0.038	0.742 ± 0.027	0.563 ± 0.030	0.758 ± 0.042	0.762 ± 0.026	0.753 ± 0.039
GigaPath	0.748 ± 0.049	0.655 ± 0.032	0.750 ± 0.036	0.576 ± 0.019	0.732 ± 0.045	0.754 ± 0.029	0.750 ± 0.043
CTransPath	0.739 ± 0.036	0.679 ± 0.042	0.738 ± 0.026	0.560 ± 0.022	0.736 ± 0.036	0.743 ± 0.041	0.736 ± 0.036
Hibou	0.750 ± 0.044	0.697 ± 0.025	0.744 ± 0.037	0.585 ± 0.038	0.722 ± 0.035	0.757 ± 0.028	0.758 ± 0.038
H-Optimus-1	0.762 ± 0.046	0.696 ± 0.039	0.744 ± 0.033	0.589 ± 0.026	0.760 ± 0.026	0.765 ± 0.035	0.748 ± 0.028
Virchow2	0.752 ± 0.048	0.686 ± 0.028	0.729 ± 0.034	0.559 ± 0.037	0.735 ± 0.045	0.753 ± 0.033	0.729 ± 0.035
CONCHv1.5	0.731 ± 0.045	0.680 ± 0.040	0.702 ± 0.035	0.582 ± 0.024	0.711 ± 0.036	0.720 ± 0.036	0.725 ± 0.034
UNI2	0.759 ± 0.044	0.672 ± 0.034	0.748 ± 0.038	0.575 ± 0.031	0.738 ± 0.037	0.760 ± 0.035	0.771 ± 0.054
CHIEF	0.738 ± 0.033	0.713 ± 0.032	0.733 ± 0.022	0.569 ± 0.039	0.750 ± 0.032	0.742 ± 0.031	0.750 ± 0.038
PRISM	0.716 ± 0.030	0.688 ± 0.043	0.702 ± 0.035	0.573 ± 0.028	0.720 ± 0.045	0.715 ± 0.031	0.715 ± 0.046
TITAN	0.725 ± 0.031	0.709 ± 0.042	0.719 ± 0.046	0.565 ± 0.026	0.740 ± 0.046	0.731 ± 0.030	0.734 ± 0.037

Abbreviations:

ADG: adult-type diffuse glioma;

CI: confidence interval;

FM: foundation model;

GBM: glioblastoma;

IDH: isocitrate dehydrogenase;

IPCW: inverse probability of censoring weighting;

MIL: multiple instance learning;

NR: not reached;

OS: overall survival;

SD: standard deviation;

SS: sum of squares;

tdAUC: time-dependent area under the curve.
